# Aniquinazolines A–D, Four New Quinazolinone Alkaloids from Marine-Derived Endophytic Fungus *Aspergillus nidulans*

**DOI:** 10.3390/md11072682

**Published:** 2013-07-23

**Authors:** Chun-Yan An, Xiao-Ming Li, Chun-Shun Li, Ming-Hui Wang, Gang-Ming Xu, Bin-Gui Wang

**Affiliations:** 1 Key Laboratory of Experimental Marine Biology, Institute of Oceanology, Chinese Academy of Sciences, Nanhai Road 7, Qingdao 266071, China; E-Mails: anchy5885@gmail.com (C.-Y.A.); lixmqd@yahoo.com.cn (X.-M.L.); lichunshun@ms.qdio.ac.cn (C.-S.L.); wangmh29@yahoo.cn (M.-H.W.); AericXu@gmail.com (G.-M.X.); 2 University of Chinese Academy of Sciences, Yuquan Road 19A, Beijing 100049, China

**Keywords:** mangrove, endophytic fungus, *Aspergillus nidulans*, secondary metabolites, bioactivity

## Abstract

Four new quinazolinone alkaloids, namely, aniquinazolines A–D (**1**–**4**), were isolated and identified from the culture of *Aspergillus nidulans* MA-143, an endophytic fungus obtained from the leaves of marine mangrove plant *Rhizophora stylosa*. The structures of the new compounds were elucidated by spectroscopic analysis, and their absolute configurations were determined on the basis of chiral HPLC analysis of the acidic hydrolysates. The structure for **1** was confirmed by single-crystal X-ray diffraction analysis. All these compounds were examined for antibacterial and cytotoxic activity as well as brine shrimp (*Artemia salina*) lethality.

## 1. Introduction

**Figure 1:**
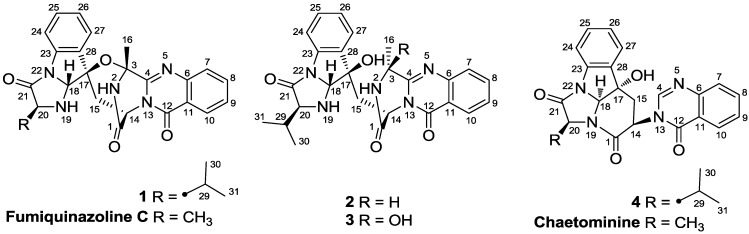
Structures of the isolated compounds **1**–**4** and reference compounds fumiquinazoline C and chaetominine.

Quinazolinone derivatives generally refer to the compounds possessing a building block of quinazolin-4-one. More than 160 naturally occurring quinazolinones have been isolated from different organisms including plants, animals and microorganisms [1]. This family of alkaloids has attracted great attention due to their diversified structures and potent biological activities such as cytotoxicity, anti-feedant, and anti-microbial activities [2,3,4,5,6,7,8,9]. It is notable that some quinazolinone derivatives biogenetically synthesized by nonribosomal peptide synthesis (NRPS) [10] were isolated from a diverse group of fungi including *Aspergillus fumigatus*, *A. flavipes*, *A. versicolor*, *Chaetomium* sp., and *Penicillium thymicola*. During our ongoing investigation for the bioactive secondary metabolites of marine-derived fungi [11,12,13,14,15], four new quinazolinone alkaloids, namely, aniquinazolines A–D (**1**–**4**) (Figure 1), were isolated and elucidated from the culture of the fungus *Aspergillus nidulans* MA-143, which was obtained from the leaves of a mangrove plant *Rhizophora stylosa*. Details of the isolation, structure elucidation, and biological activity of compounds **1**–**4** are reported herein.

## 2. Results and Discussion

### 2.1. Structure Elucidation of the New Compounds **1**–**4**

**Figure 2:**
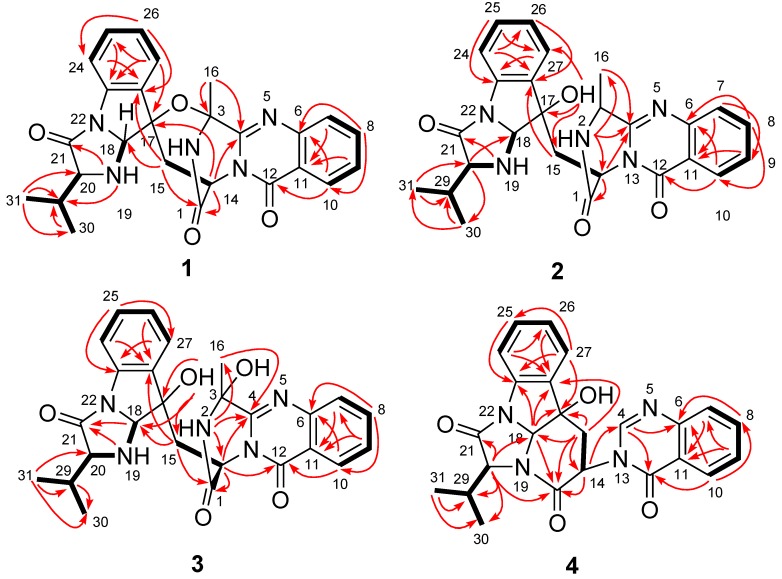
Key ^1^H–^1^H COSY (bold lines) and HMBC (red arrows) correlations of compounds **1**–**4**.

Compound **1** was obtained as a colorless crystal. Its molecular formula was determined as C_26_H_25_N_5_O_4_ on the basis of HRESIMS, with 17 degrees of unsaturation. Analysis of the ^1^H NMR spectrum of **1** revealed the resonances of four triple-doublets at δ_H_ 7.88 (td dd, *J* = 7.9, 1.3 Hz, H-8), 7.61 (td, *J* = 7.9, 0.9 Hz, H-9), 7.31 (td, *J* = 7.7, 0.9 Hz, H-25) and 7.04 (td, *J* = 7.7, 0.9 Hz, H-26), two double-doublets at δ_H_ 7.75 (dd, *J* = 7.9, 0.9 Hz, H-7) and 8.18 (dd, *J* = 7.9, 1.3 Hz, H-10), and two doublets at δ_H_ 7.38 (d, *J* = 7.7 Hz, H-24) and 7.25 (d, *J* = 7.7 Hz, H-27) (Table 1), which indicated the presence of two 1,2-disubstituted phenyl groups (C-6 to C-11 and C-23 to C-28) in **1**. Its ^13^C and DEPT NMR spectra showed signals for three methyls, one methylene, eight sp^2^ and four sp^3^ methines, as well as ten quaternary carbons including two oxygenated sp^3^ carbons and three ester/amide carbonyl carbons (Table 2). A detailed NMR data comparison with those reported for fumiquinazoline C, a quinazolinone alkaloid identified from a fungal strain of *Aspergillus fumigatus* originally derived from the marine fish *Pseudolabrus japonicus* [2], revealed that the two compounds had the same carbon skeleton. Compound **1** differed from fumiquinazoline C mainly at the presence of an isopropyl group (δ_H_ 2.09/δ_C_ 31.4, CH-29; δ_H_ 1.07/δ_C_ 17.9, CH_3_-30; and δ_H_ 1.07/δ_C_ 18.5, CH_3_-31) connected at C-20 in **1**, as it was a methyl group (δ_H_ 1.06/δ_C_ 18.7, CH_3_) in that of fumiquinazoline C. This deduction was further verified by the ^1^H–^1^H COSY correlations from H-29 to H-20 and H_3_-30/H_3_-31 as well as by the observed HMBC correlations from H_3_-31 to C-20, C-29, and C-30 and from NH-19 to C-29 as shown in Figure 2.

**Table 1:** ^1^H NMR (500MHz) data ofcompounds **1**–**4** in DMSO-*d*_6_ (δ in ppm).

Position	1 (*J* in Hz)	2 (*J* in Hz)	3 (*J* in Hz)	4 (*J* in Hz)
2-NH	9.84, br s	8.61, br s	9.49, br s	
3		4.94, q (6.5)		
4				8.30, s
7	7.75, dd (7.9, 0.9)	7.66, d (7.8)	7.70, m	7.71, dd (7.9, 0.8)
8	7.88, td (7.9, 1.3)	7.82, t (7.8)	7.86, td (7.8, 1.2)	7.87, td (7.9, 1.3)
9	7.61, td (7.9, 0.9)	7.53, t (7.8)	7.58, td (7.8, 1.2)	7.59, td (7.9, 0.8)
10	8.18, dd (7.9, 1.3)	8.12, d (7.8)	8.16, dd (7.8, 1.2)	8.18, dd (7.9, 1.3)
14	5.36, dd (5.5, 1.5)	5.58, dd (9.6, 4.3)	5.44, dd (7.4, 6.5)	4.86, dd (8.1, 5.3)
15	2.98, dd (14.0, 5.5) 1.92,dd (14.0, 1.5)	2.59, dd (14.8, 9.6) 1.83, dd (14.8, 4.3)	2.61, dd (14.5, 7.4) 2.20, dd (14.5, 6.5)	3.01, dd (14.0, 8.1) 2.77, dd (14.0, 5.3)
16	1.91, s	1.59, d (6.5)	1.76, s	
17-OH		5.67, br s	5.67, br s	5.45, br s *
18	5.70, dd (6.0, 1.5)	5.26, d (5.8)	5.31, dd (7.3, 1.5)	5.82, s
19-NH	2.67, d (5.5)	3.51, m	3.38, dd (7.3, 2.8)	
20	3.59, br d (5.5)	3.55, br s	3.53, br s	4.21, d (9.3)
24	7.38, d (7.7)	7.35, d (7.8)	7.34, d (7.6)	7.47, d (7.4)
25	7.31, td (7.7, 0.9)	7.26, t (7.8)	7.27, td (7.6, 1.0)	7.43, td (7.4, 1.2)
26	7.04, td (7.7, 0.9)	7.09, t (7.8)	7.15, td (7.6, 1.0)	7.26, td (7.4, 1.2)
27	7.25, d (7.7)	7.82, d (7.8)	7.70, m	7.54, d (7.4)
29	2.09, dq (13.4, 6.8)	1.99, m	1.98, m	2.45, m
30	1.07, d (6.8)	0.93, d (6.8)	0.91, d (6.6)	1.15, d (6.7)
31	1.07, d (6.8)	0.96, d (6.8)	0.92, d (6.6)	1.12, d (6.7)

* This exchangeable proton was detected in acetone-*d*_6_.

**Table 2:** ^13^C NMR (125MHz) data of compounds **1**–**4** in DMSO-*d*_6_ (δ in ppm).

Position	1	2	3	4
1	168.6, C	168.9, C	169.7, C	164.8, C
3	84.2, C	48.5, CH	84.4, C	
4	151.5, C	153.3, C	150.3, C	146.7, CH
6	146.6, C	146.6, C	146.0, C	147.5, C
7	127.8, CH	126.9, CH	127.3, CH	127.1, CH
8	134.6, CH	134.4, CH	134.6, CH	134.6, CH
9	127.5, CH	126.7, CH	127.4, CH	127.3, CH
10	126.4, CH	126.4, CH	126.4, CH	126.1, CH
11	120.5, C	120.0, C	120.1, C	121.5, C
12	158.8, C	160.2, C	160.5, C	159.7, C
14	52.7, CH	51.8, CH	52.6, CH	55.6, CH
15	33.7, CH_2_	35.5, CH_2_	39.1, CH_2_	34.1, CH_2_
16	23.7, CH_3_	16.5, CH_3_	20.3, CH_3_	
17	86.3, C	80.1, C	80.3, C	73.8, C
18	88.4, CH	88.1, CH	88.1, CH	83.7, CH
20	69.0, CH	69.1, CH	69.0, CH	69.3, CH
21	171.5, C	172.1, C	172.6, C	173.8, C
23	136.6, C	137.2, C	137.7, C	140.2, C
24	114.3, CH	115.1, CH	115.2, CH	114.2, CH
25	129.7, CH	128.8, CH	128.6, CH	130.0, CH
26	125.1, CH	124.6, CH	124.6, CH	125.1, CH
27	126.2, CH	125.4, CH	125.2, CH	124.5, CH
28	137.4, C	138.7, C	138.8, C	134.9, C
29	31.4, CH	31.0, CH	31.0, CH	28.2, CH
30	17.9, CH_3_	17.6, CH_3_	17.4, CH_3_	18.6, CH_3_
31	18.5, CH_3_	18.6, CH_3_	18.5, CH_3_	20.1, CH_3_

**Figure 3:**
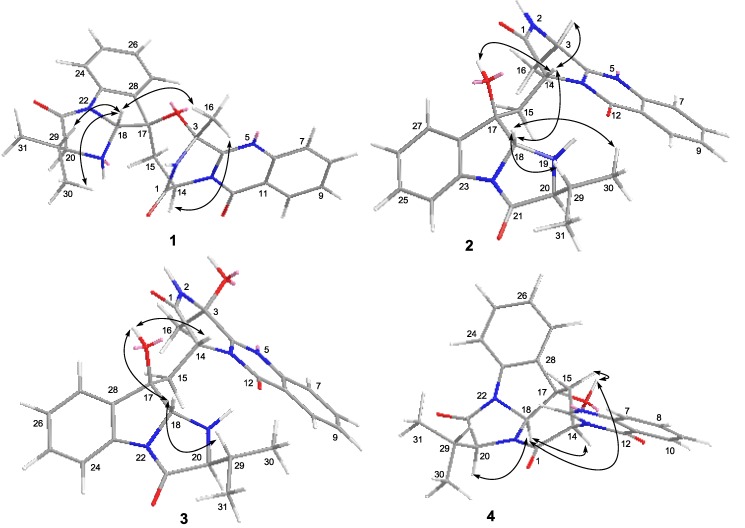
NOESY correlations of compounds **1**–**4**.

**Figure 4:**
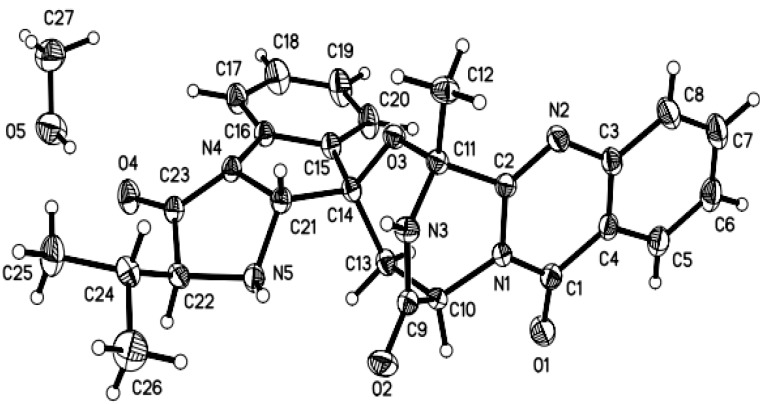
X-ray structure of compound **1** (Note: A different numbering system is used for the structure in the text).

**Figure 5:**
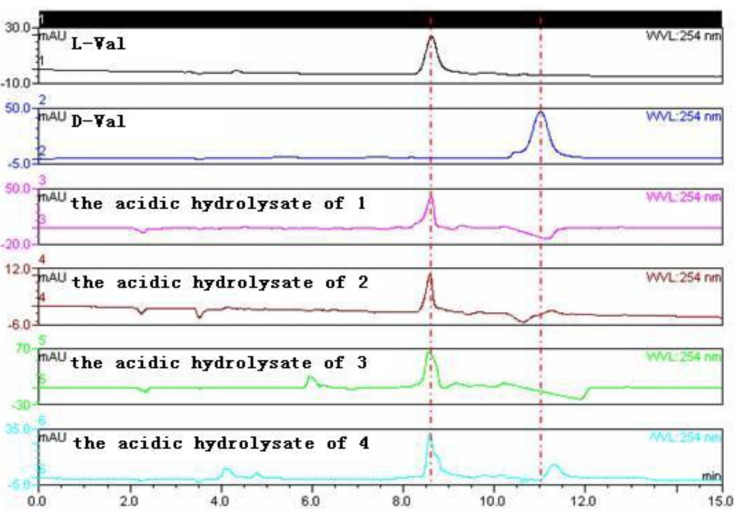
Chiral HPLC analysis of the amino acid derivatives of compounds **1**–**4**.

**Figure 6:**
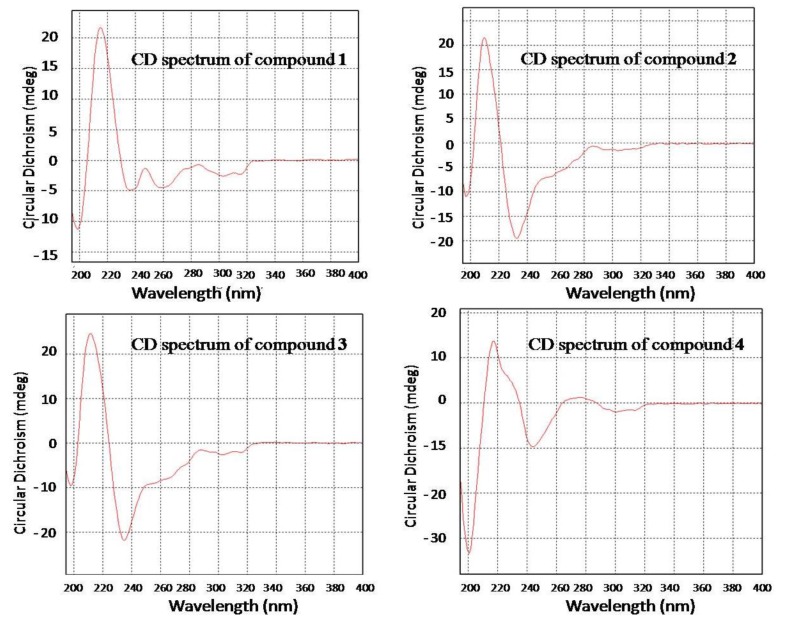
Electronic Circular Dichroism (ECD) spectra of compounds **1**–**4**.

The relative configuration of compound **1** was determined by NOESY spectrum and by the X-ray experiment. The NOE correlations from H-16 to H-14 and H-18 as well as from H-18 to H-29 indicated the same orientation of these protons (Figure 3). The structure and relative configuration of compound **1** were confirmed by the single crystal X-ray diffraction analysis (Figure 4). The absolute configuration of **1** was deduced by chiral HPLC analysis of the degradation products of the acidic hydrolysates. The HPLC profile of the hydrolysates was compared with that of authentic standard to establish the configuration of the amino acid-derived unit as l-valine (Figure 5), corresponding to the *S*-configuration for C-20. The absolute configuration of compound **1** was therefore assigned to be 3*R*, 14*R*, 17*R*, 18*R*, and 20*S*, which was identical to that of fumiquinazoline C [2]. The ECD (Electronic Circular Dichroism) spectrum of **1** showed negative Cotton Effect (CE) at 199, 238, 258, and 303 nm, and positive CE at 216 nm (Figure 6). The structure of **1** was thus elucidated and it was named as aniquinazoline A.

Compound **2** was obtained as yellowish solid. The HRESIMS experiment established its molecular formula C_26_H_27_N_5_O_4_ (16 degrees of unsaturation), with two more *H*-atom than that of **1**. The ^1^H and ^13^C NMR data of **2** were very similar to those of **1** (Table 1, Table 2), indicating that they shared the same carbon skeleton. However, the oxygenated sp^3^ quaternary carbon at δ_C_ 84.2 (C-3) in the ^13^C NMR spectrum of **1** disappeared in that of **2**. Instead, signals for a methine group (δ_H_ 4.94/δ_C_ 48.5, CH-3) were observed in the NMR spectra of **2** (Table 1, Table 2). Accordingly, the singlet methyl signal at δ_H_ 1.91 (H_3_-16) in the ^1^H NMR spectrum of **1** was replaced by a doublet methyl group at δ_H_ 1.59 (*J* = 6.5 Hz) in that of **2**, and an exchangeable OH proton at δ_H_ 5.67 (br s, 17-OH) was observed (Table 1). This OH group was attached at C-17 on the basis of HMBC correlations from the OH proton to C-15 (δ_C_ 35.5, CH_2_), C-17 (δ_C_ 80.1, C), and C-27 (δ_C_ 125.4, CH) (Figure 2). The ^1^H–^1^H COSY and HMBC correlations shown in Figure 2 confirmed the planar structure of **2**. The relative configuration of compound **2** was determined by NOESY spectrum as well as by comparing the ^1^H-NMR *J*-values with that of fumiquinazolines A and B [2]. The observed NOE correlations from H-18 to H-14 and H-29 and from H-14 to H-3 and 17-OH, indicated them on the same face of the molecule (Figure 3). In contrast to compound **1**, no NOE correlations could be observed from H-16 to H-14 and H-18. Instead, NOE correlation from H-14 to H-3 was observed in the NOESY spectrum of **2** (see Figure 3 as well as Figure S12b in the Supplementary Materials). In the reference report, a large coupling constant (*J* = 4.9 Hz) for H-3 and NH-2 was observed in fumiquinazoline B, whereas its C-3 epimer, fumiquinazoline A, showed a very small coupling constant (*J* = 0.3 Hz) [2]. In the case of compound **2**, the proton NH-2 was observed as a broad singlet (Table 1), which means the coupling constant between H-3 and NH-2 was very small, thus suggesting the configuration at C-3 is close to that of funiquinazoline A. The above data suggested that the C_3_–O–C_17_ ether ring in **1** was opening in that of **2**, and the configuration of C-3 changed during the ring-opening process. To substantiate this, compound **1** was reduced with NaBH_4_ in THF at room temperature for 40 min and the resulting product was revealed to be identical to **2** by co-TLC analysis. The ^1^H-NMR and ^1^H–^1^H COSY as well as NOESY spectra also showed that the reduced product of **1** was the same as that of compound **2** (see Supplementary Material). However, it was described in the reference that reduction of fumiquinazoline C yielded fumiquinazoline A without C-3 configuration change [2], and this differs from our observation as discussed above. Unfortunately, no NOESY experiment was performed and described for the reducing product of fumiquinazoline C [2], which would have helped give solid evidence to clarify the problem. Based on the above data, the relative configurations between C-3 and C-14 of fumiquinazoline A [2] should be reconsidered.

The absolute configuration of C-20 in compound **2** was also assigned as *S* based on the chiral HPLC analysis of its acidic hydrolysates (Figure 5) and the absolute configurations of other chiral centers in **2** were therefore assigned as 3*R*, 14*R*, 17*R*, and 18*R*. The structure of **2** was thus elucidated and named as aniquinazoline B.

Compound **3** was obtained as a yellowish solid. Its molecular formula was determined to be C_26_H_27_N_5_O_5_ (16 degrees of unsaturation) on the basis of HRESIMS data, with one more *O*-atom than that of **2**. The assignments of the NMR data of **3** (Table 1, Table 2) matched well with that of the corresponding signals for **2**, except for the presence of an OH group at C-3 in **3**, which was consistent with the difference in molecular formula. Accordingly, the non-oxygenated CH signals resonated at δ_H_ 4.94/δ_C_ 48.5 (CH-3) in the NMR spectra of **2** and disappeared in that of **3**. Instead, an oxygenated quaternary carbon at δ_C_ 84.4 (C-3) was observed in the ^13^C NMR spectrum of **3** (Table 2). Correspondingly, the methyl doublet signal at δ_H_ 1.59 (*J* = 6.5 Hz, H_3_-16) in the ^1^H NMR spectrum of **2** was replaced by a downfield singlet at δ_H_ 1.76 (H_3_-16) in that of **3** (Table 1). The ^1^H–^1^H COSY and HMBC correlations shown in Figure 2 confirmed the planar structure of **3**.

The relative configuration of compound **3** was determined by NOESY spectrum. The NOE correlations from H-18 to H-29 and 17-OH and from 17-OH to H-14 revealed these protons were on the same face of the molecule (Figure 3). The relative configuration at C-3 was assigned as being the same as that of **2** from the biogenetic point of view. The absolute configuration of C-20 was also determined as *S* by the chiral HPLC analysis of the acidic hydrolysates of **3** (Figure 5). Consequently, the absolute configuration of compound **3** was assigned as 3*S*, 14*R*, 17*R*, 18*R*, and 20*S*. The very similar ECD spectra for compounds **2** and **3** (Figure 6) supported the above assignment. The structure of compound **3** was thus determined and was named aniquinazoline C.

Compound **4** was isolated as a yellowish solid. Its molecular formula was determined as C_24_H_22_N_4_O_4_ by HRESIMS data. Detailed comparison of NMR data of compound **4** (Table 1, Table 2) with those of chaetominine, a cytotoxic alkaloid produced by endophytic *Chaetomium* sp. IFB-E015 originally isolated from the apparently healthy *Adenophora axilliflora* leaves [5], suggested that they shared the same carbon skeleton. The main difference was that the methyl group at C-20 in chaetominine was replaced by an isopropyl group in **4**. This deduction was verified by ^1^H–^1^H COSY and HMBC correlations as shown in Figure 2. The relative configuration of **4** was assigned by NOESY spectrum. The correlations from H-18 to H-14, 17-OH and H-20 revealed their same orientation on the molecule (Figure 3). The absolute configuration of C-20 was assigned as *S* on the basis of the chiral HPLC analysis of the acidic degradation product of **4** (Figure 5). Thus the absolute configuration of compound **4** was determined as 14*R*, 17*S*, 18*S*, and 20*S*, and it was named as aniquinazoline D.

### 2.2. Biological Activities of the Isolated Compounds

The isolated compounds **1**–**4** were examined for brine shrimp toxicity, antitumor, and antibacterial activities. Compounds **1**–**4** showed potent lethality against brine shrimp with LD_50_ values of 1.27, 2.11, 4.95 and 3.42 μΜ, respectively, which were stronger than that of the positive control colchicine (with LD_50_ value of 88.4 μΜ). Compounds **1**–**4** were also evaluated for antitumor and antibacterial activity, but none of them displayed inhibitory activity against four cell lines (BEL-7402, MDA-MB-231, HL-60, and K562) and two bacteria (*Escherichia coli* and *Staphyloccocus aureus*).

## 3. Experimental Section

### 3.1. General

The melting point was determined with an SGW X-4 micro-melting-point apparatus. The optical rotations were measured on an Optical Activity A-55 polarimeter. UV data were obtained on a Lengguang Gold S54 spectrophotometer. The ^1^H, ^13^C, and 2D NMR spectroscopic data were acquired on Bruker Advance 500 spectrometers. Mass spectra were measured on a VG Autospec 3000 mass spectrometer. HPLC analysis was performed on a Dionex HPLC System equipped with a P680 pump, an ASI-100 automated sample injector, a TCC-100 column oven, a UV-DAD 340U detector, and a Dionex Acclaim ODS column (4.6 × 250 mm, 5 μm). Semi-preparative HPLC was operated on a Dionex UltiMate U3000 system using an Agilent Prep RP-18 column (21.2 × 250 mm, 10 μm) with UV detection. A Phenomenex-Chirex 3126 *N*,*S*-dioctyl-(d)-penicillamine column (250 × 4.60 mm, 5 μm) was used for chiral HPLC analysis. Column chromatography (CC) was performed with silica gel (200–300 mesh, Qingdao Marine Chemical Factory), Lobar LiChroprep RP-18 (40–63 μm; Merck), and Sephadex LH-20 (18–110 μm, Merck).

### 3.2. Fungal Material

The fungus *Aspergillus nidulans* MA-143 was isolated from the leaves of marine mangrove plant *Rhizophora stylosa*. The fungus grew slowly on potato dextrose agar (PDA) plate and turned from white to green mycelia within 5 days. The fungus was identified by sequence analysis of the ITS region of its rDNA as described previously [16] and the sequence data obtained from the fungus have been deposited in GenBank with accession number JQ839285. The strain is preserved at the Key Laboratory of Experimental Marine Biology, Institute of Oceanology, Chinese Academy of Sciences.

### 3.3. Fermentation

The fermentation was statically carried out in liquid potato-dextrose broth medium (PDB) (1000 mL seawater, 20 g glucose, 5 g peptone, 3 g yeast extract, pH 6.5–7.0, liquid medium/ﬂask = 300 mL) in 1 L Erlenmeyer ﬂasks for 30 days at room temperature.

### 3.4. Extraction and Isolation

The fermented whole broth (100 ﬂasks) was ﬁltered through cheesecloth to separate the culture broth and mycelia, which were extracted exhaustively with EtOAc and MeOH, respectively. Since the chemical profiles of the two extracts were almost identical, they were combined and concentrated to afford the crude extract (31.0 g) for further separation. The crude extract was subjected to silica gel vacuum liquid chromatography (VLC) eluting with mixed solvents of increasing polarity (petroleum ether-EtOAc, 5:1 to 1:1, and then CHCl_3_–MeOH, 20:1 to 0:1) to yield 7 fractions (Fr.1 to Fr.7). Fraction 2 (11.0 g) was further separated to seven subfractions (Fr.2.1–2.7) by CC on silica gel eluted with CHCl_3_–MeOH (100:1 to 5:1). Fr.2.1 (7.0 g) was subjected to CC on Lobar LiChroprep C_18_ eluting with MeOH-H_2_O gradient (1:9 to 1:0) and then further purified by semi-preparative HPLC (MeOH–H_2_O 7:3, 16 mL/min) to afford **2** (*t*_R_ = 14.6 min, 5.2 mg). Fr.2.2 (3.0 g) was subjected to CC on silica gel eluted with CHCl_3_–MeOH (100:1 to 5:1) and Sephadex LH-20 (petroleum ether–CHCl_3_–MeOH, 5:5:1), and then further purified by semi-preparative HPLC (MeOH–H_2_O 7:3, 16 mL/min) to obtain **1** (*t*_R_ = 15.8 min, 20.0 mg), **3** (*t*_R_ = 13.5 min, 5.5 mg), and **4** (*t*_R_ = 10.5 min, 7.7 mg).

Aniquinazoline A (**1**): colorless crystal; mp 243–245 °C; [α]^20^_D_: −13 (*c* 0.30, MeOH); UV (MeOH) λ_max_ (log ε) 200 (4.41), 218 (4.26), 245 (3.97), 270 (3.82), 297 (3.32), 305 (3.37) nm; CD λ_max_ (Δε) 199 (−11.33), 216 (+21.60), 238 (−4.92), 258 (−4.52), 303 (−2.63) nm; ^1^H and ^13^C NMR data, see Table 1, Table 2; ESIMS *m/z* 472 [M + H]^+^; HRESIMS *m/z* 472.1982 [M + H]^+^ (calcd for C_2__6_H_2__6_N_5_O_4_^+^, 472.1985, Δ 0.3 ppm).

Aniquinazoline B (**2**): yellowish solid; [α]^20^_D_: −118 (*c* 0.28, MeOH); UV (MeOH) λ_max_ (log ε) 202 (4.46), 225 (4.33), 243 (4.03), 304 (3.35) nm; CD λ_max_ (Δε) 197 (−10.99), 210 (+21.55), 233 (−19.63), 304 (−1.59) nm; ^1^H and ^13^C NMR data, see Table 1, Table 2; ESIMS *m/z* 474 [M + H]^+^; HRESIMS *m/z* 474.2143 [M + H]^+^ (calcd for C_2__6_H_2__8_N_5_O_4_^+^, 474.2141, Δ 0.2 ppm).

Aniquinazoline C (**3**): yellowish solid; [α]^20^_D_: −19 (*c* 0.28, MeOH); UV (MeOH) λ_max_ (log ε) 200 (4.63), 214 (4.51), 246 (4.14), 271 (4.01), 290 (3.70), 301 (3.61) nm; CD λ_max_ (Δε) 199 (−9.37), 212 (+24.53), 235 (−21.94), 303 (−2.65) nm; ^1^H and ^13^C NMR data, see Table 1, Table 2; ESIMS *m/z* 490 [M + H]^+^; HRESIMS *m/z* 490.2084 [M + H]^+^ (calcd for C_2__6_H_2__8_N_5_O_5_^+^, 490.2090, Δ 0.6 ppm).

Aniquinazoline D (**4**): yellowish solid; [α]^20^_D_: −33 (*c* 0.37, MeOH); UV (MeOH) λ_max_ (log ε) 200 (6.06), 218 (5.91), 246 (5.61), 268 (5.52), 298 (4.98), 312 (4.92) nm; CD λ_max_ (Δε) 201 (−33.36), 218 (+13.62), 244 (−9.78), 276 (+1.17), 301 (−2.08) nm; ^1^H and ^13^C NMR data, see Table 1, Table 2; ESIMS *m/z* 431 [M + H]^+^; HRESIMS *m/z* 431.1710 [M + H]^+^ (calcd for C_24_H_2__3_N_4_O_4_^+^, 431.1719, Δ 0.9 ppm).

### 3.5. X-ray Crystallographic Analysis of Compounds **1**

All crystallographic data [17] were collected on a Bruker Smart-1000 CCD diffractometer equipped with a graphite-monochromatic Mo-*K*α radiation (λ = 0.71073 Å) at 298(2) K. The data were corrected for absorption by using the program SADABS [18]. The structure was solved by direct methods with the SHELXTL software package [19]. All non-hydrogen atoms were refined anisotropically. The H atoms were located by geometrical calculations, and their positions and thermal parameters were fixed during the structure refinement. The structure was refined by full-matrix least-squares techniques [20].

Crystal data for compound **1**: C_27_H_29_N_5_O_5_, F.W. = 503.55, one molecule containing a CH_3_OH solvent molecule in the unit, monoclinic space group P2(1), unit cell dimensions *a* = 11.3860(5) Å, *b* = 7.8898(3) Å, *c* = 13.6963(5) Å, *V* = 1230.18(8) Å^3^, α = γ = 90°, β = 91.044(3)°, *Z* = 2, *d*_calcd_ = 1.359 mg/m^3^, crystal dimensions 0.45 × 0.40 × 0.32 mm, μ = 0.096 mm^−1^, *F*(000) = 532. The 7506 measurements yielded 4172 independent reflections after equivalent data were averaged, and Lorentz and polarization corrections were applied. The final refinement gave *R*_1_ = 0.0332 and w*R*_2_ = 0.0778[*I* > 2σ(*I*)].

### 3.6. Amino Acid Analysis

Compounds **1** (1.3 mg), **2** (0.9 mg), **3** (0.8 mg), and **4** (1.1 mg) were each solved in 10 mL 6 N HCl and heated at 110 °C for 24 h in sealed tubes. The solutions were then evaporated to dryness under reduced pressure. Each sample, including the standard amino acid l-val and d-val, was solved in 1 mL of eluting solvent (2 mM CuSO_4_·5H_2_O, with 5 mL CH_3_CN in every 100 mL solvent) and was concentrated at a speed of 12,000 rpm. Chiral HPLC analysis was carried out using a Phenomenex-Chirex-3126 column at 254 nm with flow rate 1.0 mL/min at 40 °C. The HPLC analysis showed that the products of acidic hydrolysis of **1**–**4** contained the identical amino acid, which have the same retention time as that of the standard l-val. The results established the chiral center of the valine moiety in the structure of the four compounds to be *S*-conﬁguration (Figure 5).

### 3.7. Reduction of Compound **1**

Compound **1** (6.8 mg) was dissolved in 1.0 mL THF (tetrahydrofuran), and NaBH_4_ (5.0 mg) was added to the solution. The mixture was stirred at room temperature for 40 min and then concentrated under reduced pressure. The residue was diluted with water, extracted by CH_2_Cl_2_ and then evaporated. The reducing product (1.5 mg) was obtained from the extract by preparative TLC (CHCl_3_–MeOH, 30:1). Compound **2** and the reducing product were evidenced to be identical on the basis of co-TLC and NMR data analysis (see Supplementary Material, Figures S28–S30).

### 3.8. Brine Shrimp Toxicity

Brine shrimp (*Artemia salina*) toxicity of the isolated compounds was determined as described previously [21]. Colchicine was used as a positive control.

### 3.9. Cytotoxicity Assay

The cytotoxic activity against BEL-7402 (human hepatocellular carcinoma), MDA-MB-231 (human breast carcinoma), HL-60 (human promyelocytic leukemia cells), and K562 (human acute myelocytic leukemia cell line) cell lines were determined according to previously reported methods [22].

### 3.10. Antibacterial Assay

Antibacterial assay against *E. coli* and *S. aureus* was carried out using the well diffusion method [23]. Chloromycetin was used as a positive control.

## 4. Conclusions

Four new quinazolinone alkaloids (**1**–**4**) were isolated from the mangrove-derived endophytic fungus *Aspergillus nidulans* MA-143. The structures and relative configurations were determined on the interpretation of the NMR data and the absolute configuration of all compounds was determined by chiral HPLC analysis of the acid hydrolysates. Compounds **1**–**4** exhibited potent brine shrimp toxicity with LD_50_ values of 1.27, 2.11, 4.95 and 3.42 μΜ, respectively.

## Supplementary Files

Supplementary File 1 Supplementary Information (PDF, 1503 KB)
