# Supplementary material for: Aniquinazolines A–D, Four New Quinazolinone Alkaloids from Marine-Derived Endophytic Fungus Aspergillus nidulans
Source: Mar Drugs. 2013 Jul 23;11(7):2682–94. doi: 10.3390/md11072682 (PMC3736446; doi:10.3390/md11072682)

Supplementary Information

Table of Content

Figure S1. ^{1}H spectrum of compound **1**.
Figure S2. ^{13}C and DEPT spectra of compound **1**.
Figure S3. ^{1}H–^{1}H COSY spectrum of compound **1**.
Figure S4. HSQC spectrum of compound **1**.
Figure S5. HMBC spectrum of compound **1**.
Figure S6a. NOESY spectrum of compound **1**.
Figure S6b. An enlarged area of the NOESY spectrum of compound **1**.
Figure S7. ^{1}H spectrum of compound **2**.
Figure S8. ^{13}C and DEPT spectra of compound **2**.
Figure S9. ^{1}H–^{1}H COSY spectrum of compound **2**.
Figure S10. HSQC spectrum of compound **2**.
Figure S11. HMBC spectrum of compound **2**.
Figure S12a. NOESY spectrum of compound **2**.
Figure S12b. An enlarged area of the NOESY spectrum of compound **2**.
Figure S13. NOESY spectrum of compound **2** (recorded with different parameters).
Figure S14. ^{1}H spectrum of compound **3**.
Figure S15. ^{13}C and DEPT spectra of compound **3**.
Figure S16. ^{1}H–^{1}H COSY spectrum of compound **3**.
Figure S17. HSQC spectrum of compound **3**.
Figure S18. HMBC spectrum of compound **3**.
Figure S19. NOESY spectrum of compound **3**.
Figure S20. NOESY spectrum of compound **3** (recorded with different parameters).
Figure S21. NOESY spectrum of compound **3** (recorded with different parameters).
Figure S22. ^{1}H spectrum of compound **4**.
Figure S23. ^{13}C and DEPT spectra of compound **4**.
Figure S24. ^{1}H–^{1}H COSY spectrum of compound **4**.
Figure S25. HSQC spectrum of compound **4**.
Figure S26. HMBC spectrum of compound **4**.
Figure S27. NOESY spectrum of compound **4**.
Figure S28. ^{1}H spectrum of the reducing product of compound **1**.
Figure S29. ^{1}H–^{1}H COSY spectrum of the reducing product of compound **1**.
Figure S30. NOESY spectrum of the reducing product of compound **1**.

Figure S1. ^{1}H spectrum of compound **1**.

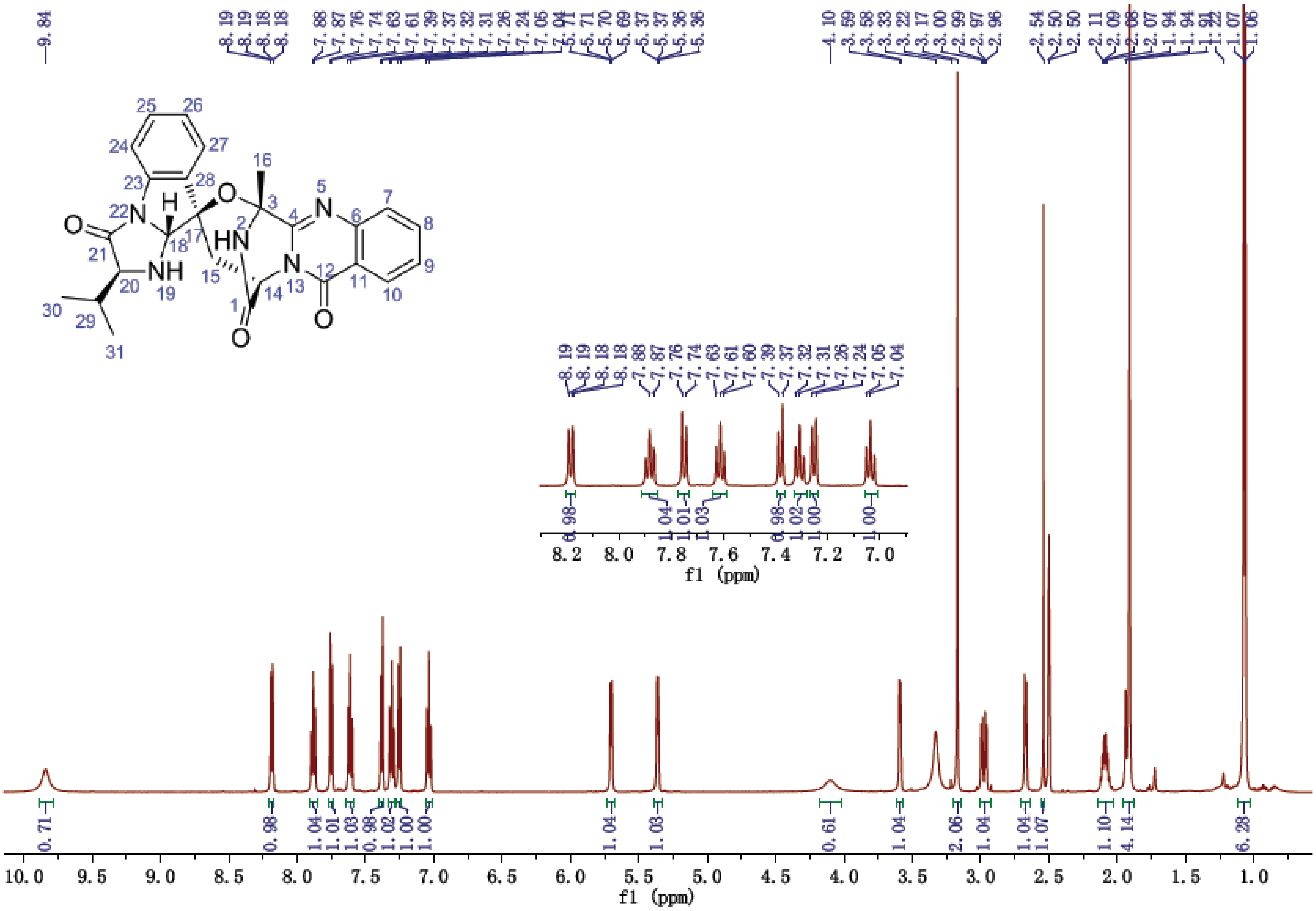

Figure S2. ^{13}C and DEPT spectra of compound **1**.

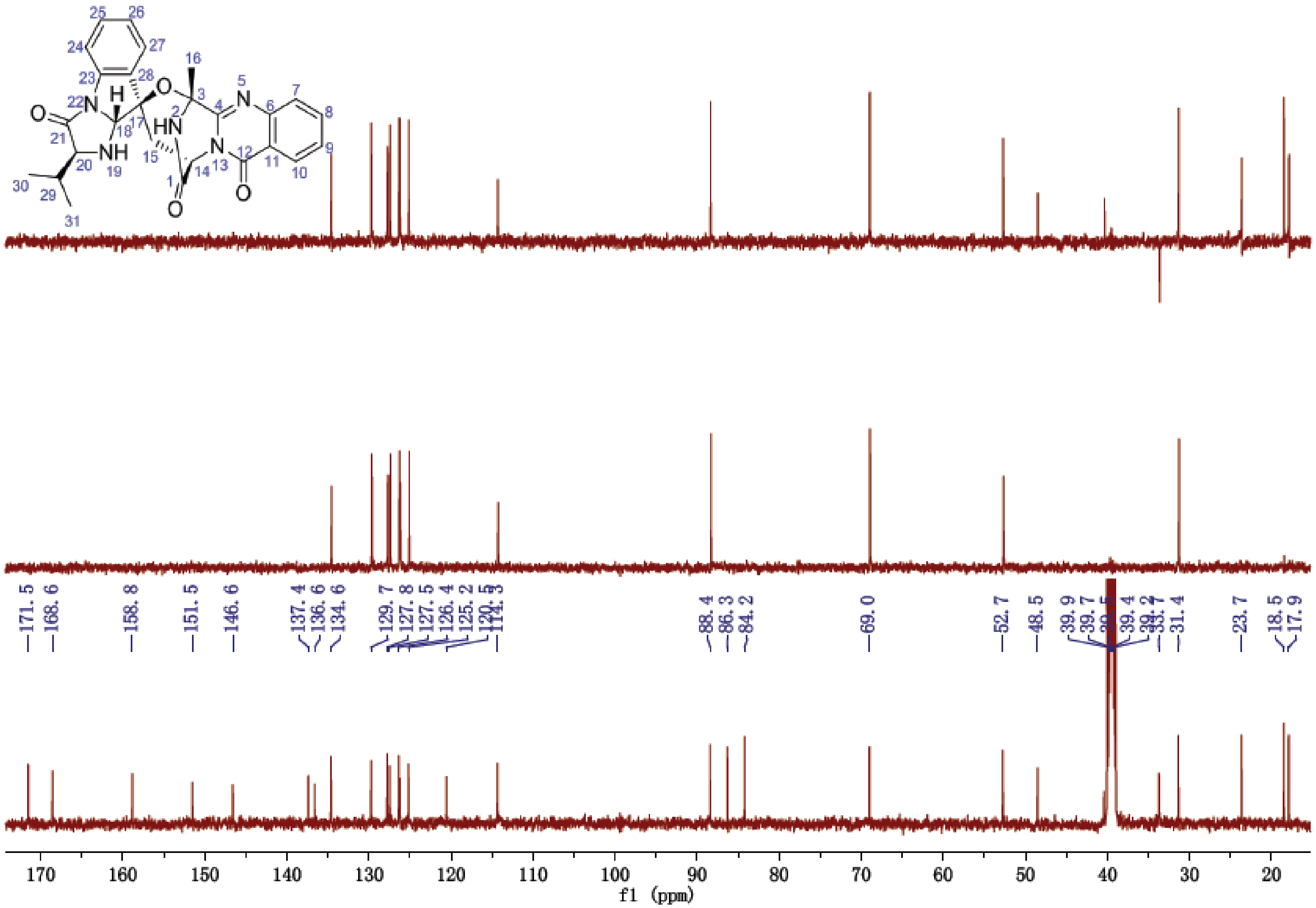

Figure S3. $^{1}H–^{1}H$ COSY spectrum of compound **1**.

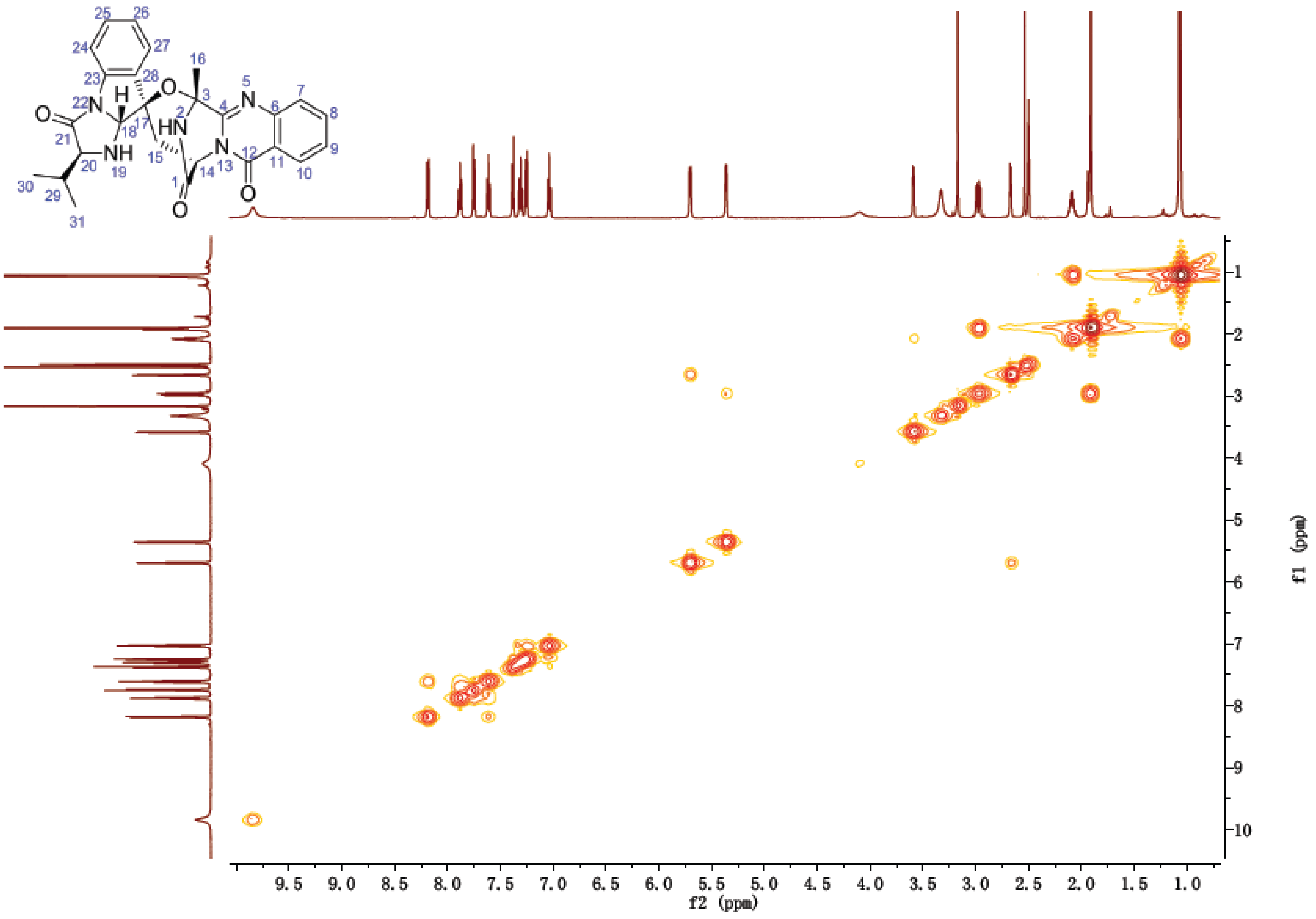

Figure S4. HSQC spectrum of compound **1**.

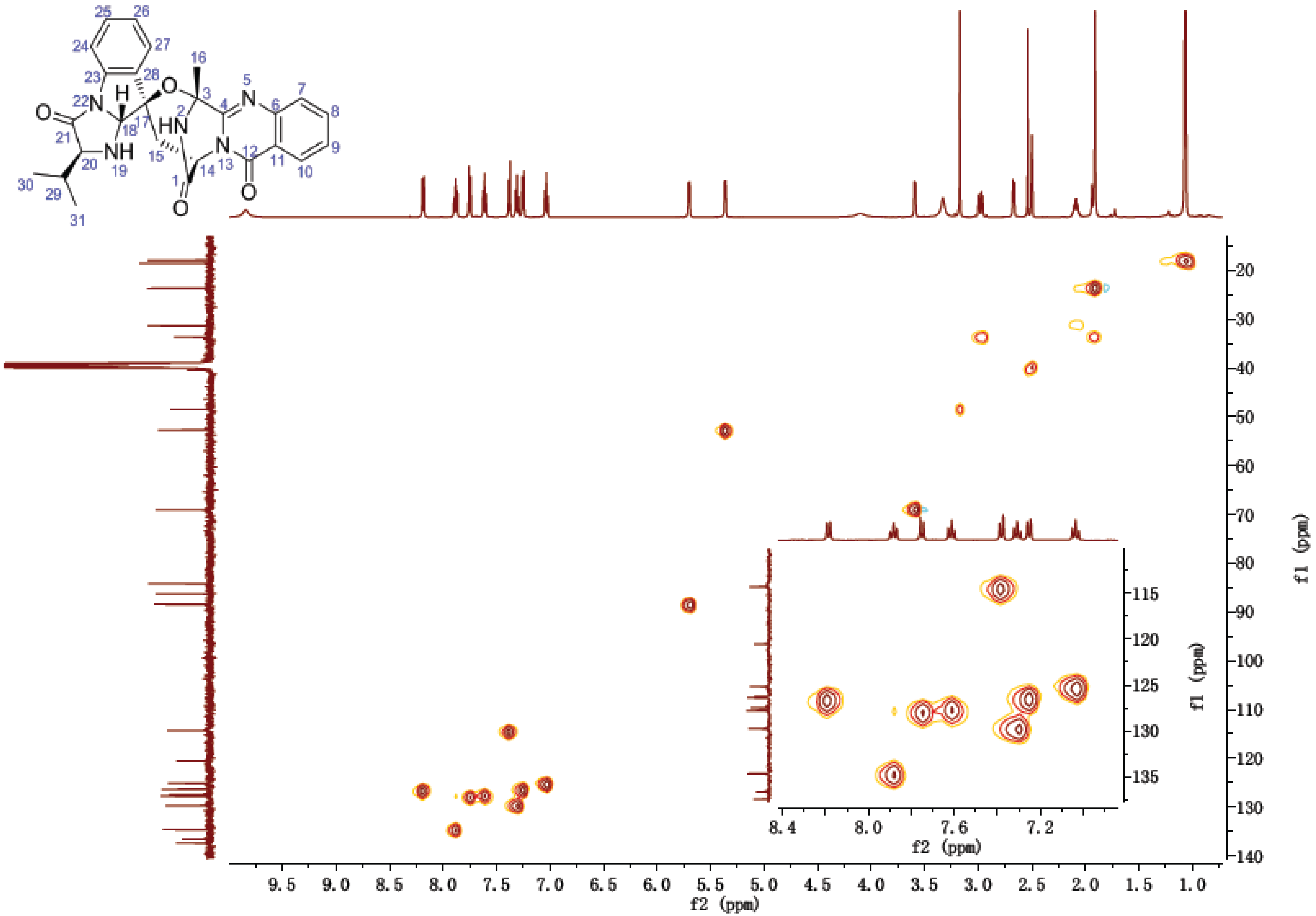

Figure S5. HMBC spectrum of compound **1**.

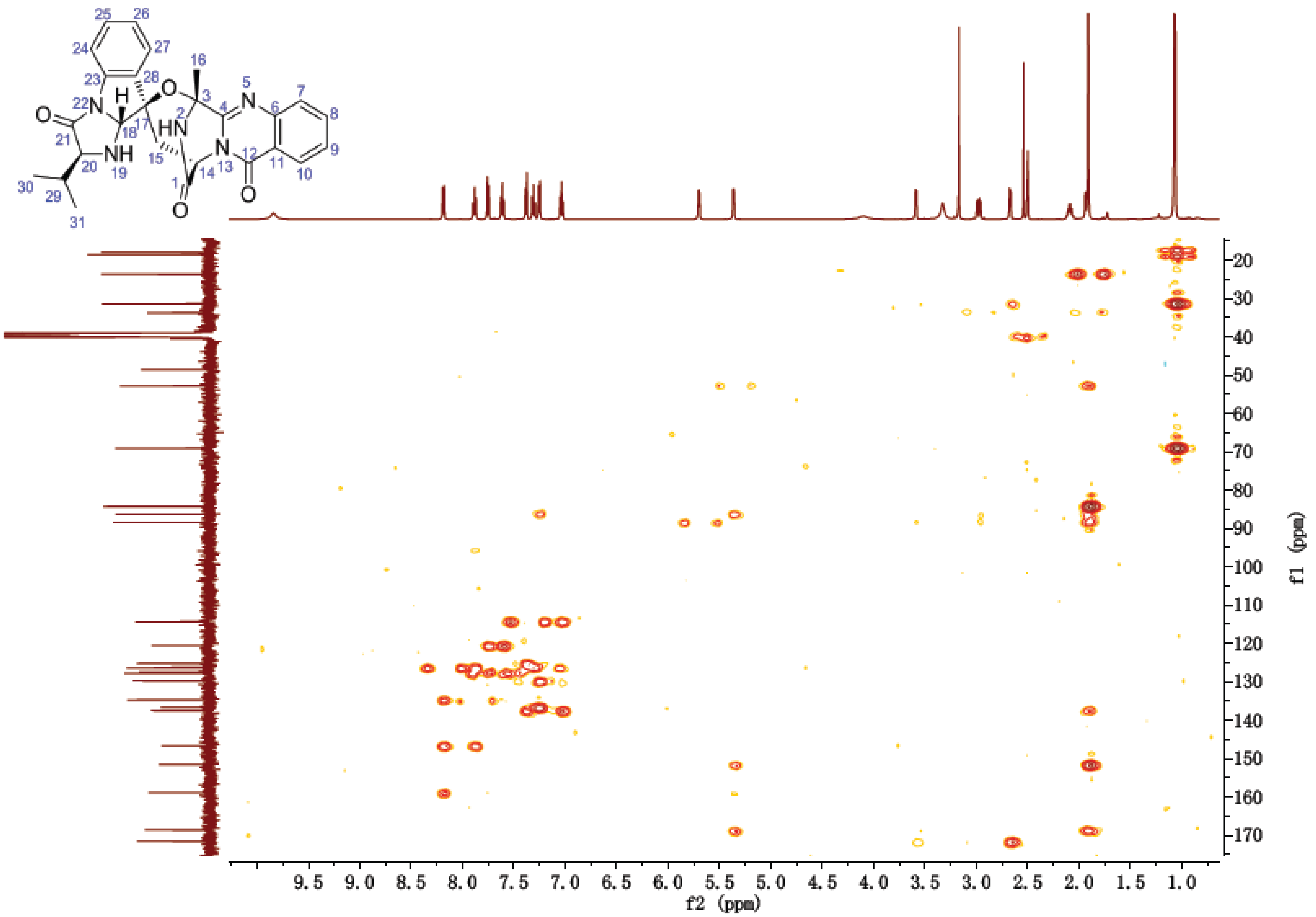

Figure S6a. NOESY spectrum of compound **1**.

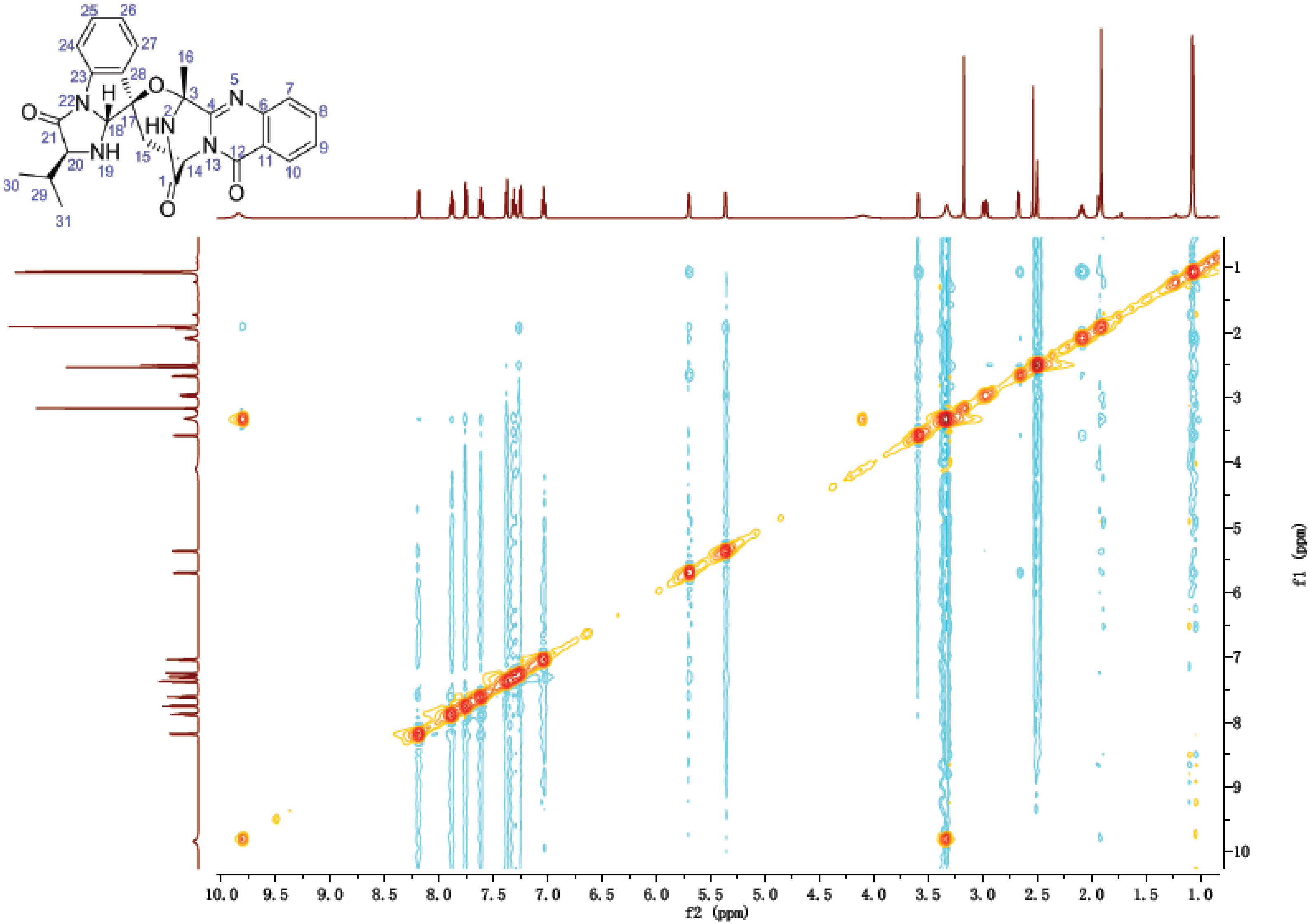

Figure S6b. an enlarged area of the NOESY spectrum of compound **1**.

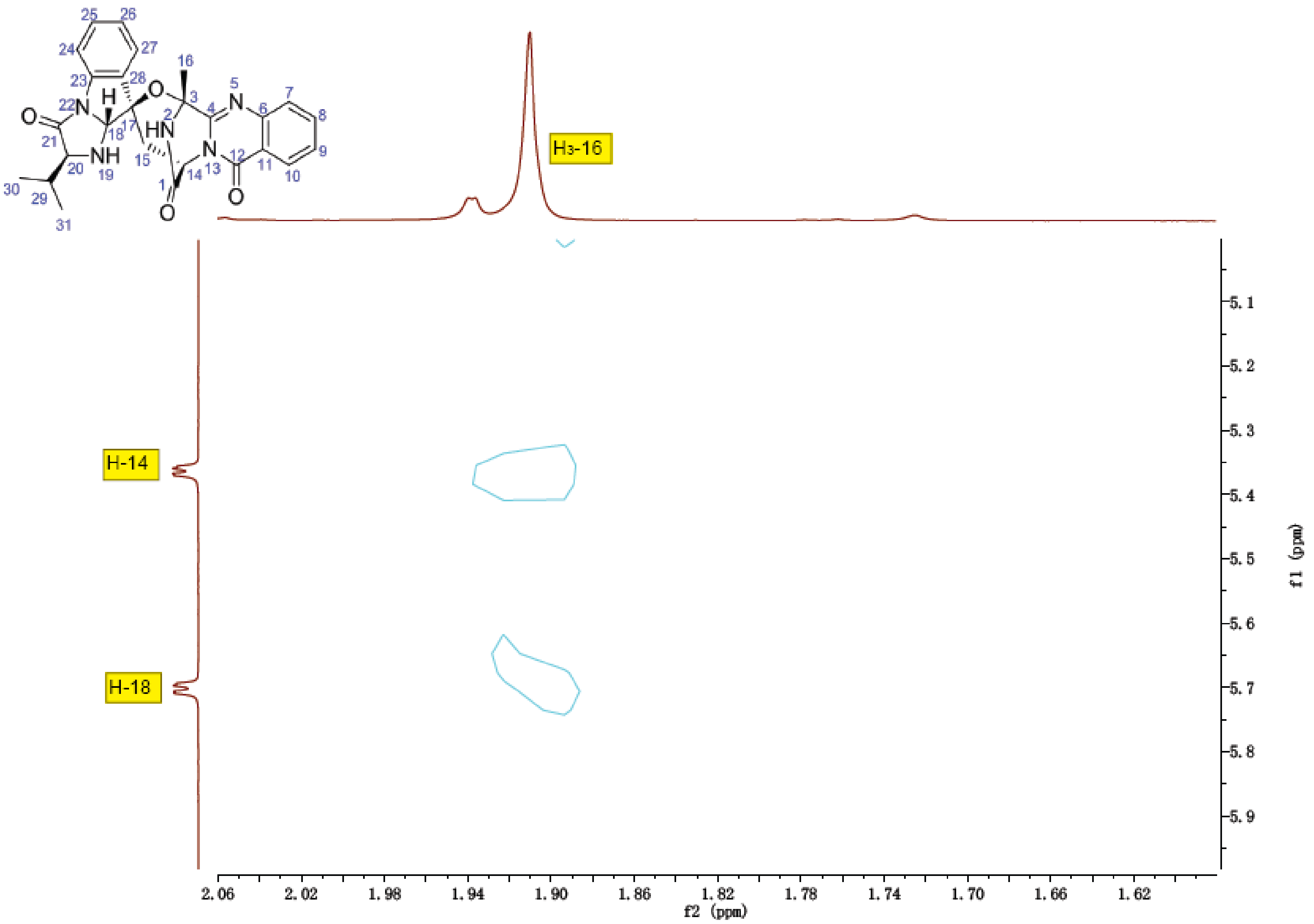

Figure S7. ^{1}H spectrum of compound **2**.

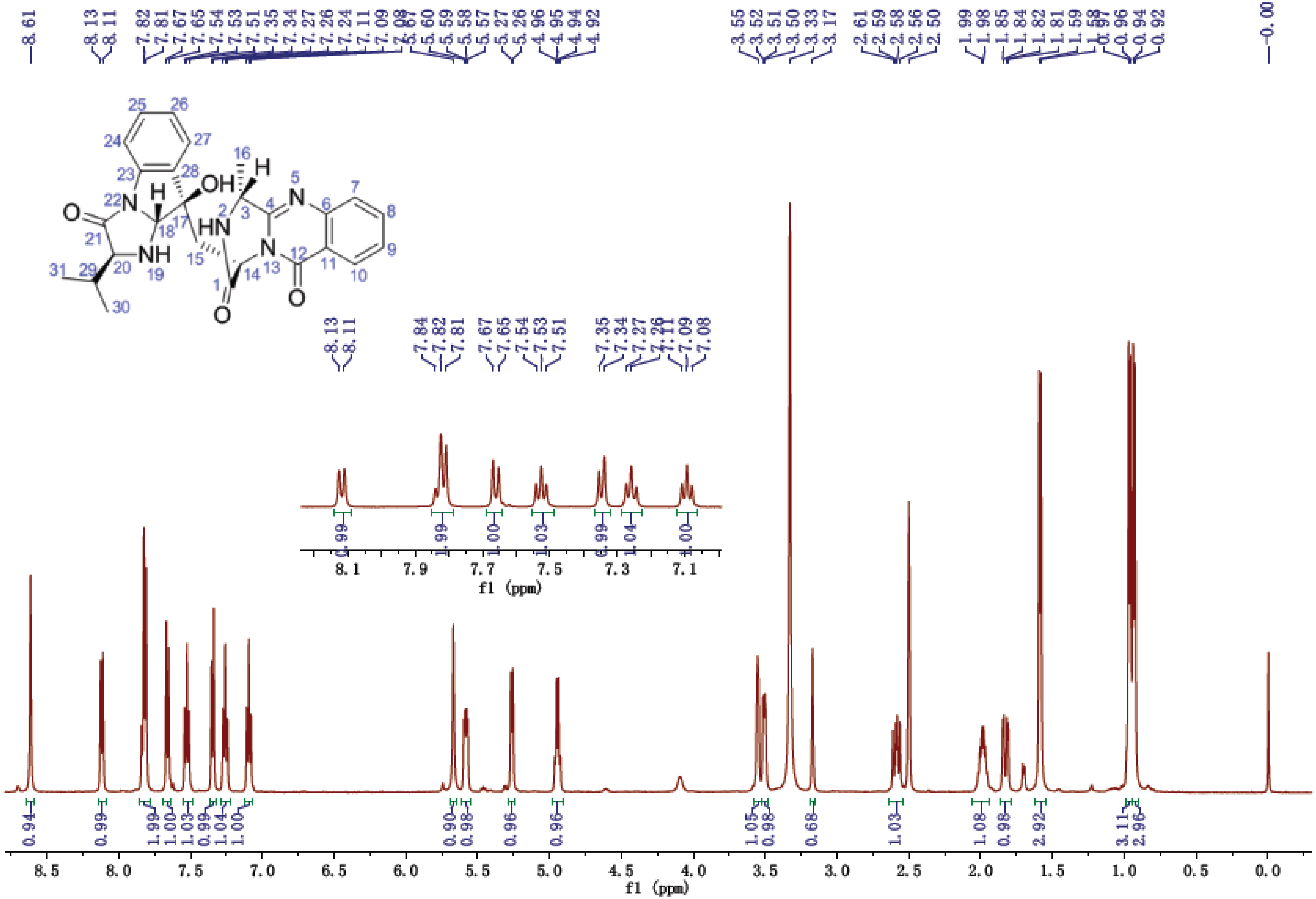

Figure S8. ^{13}C and DEPT spectra of compound **2**.

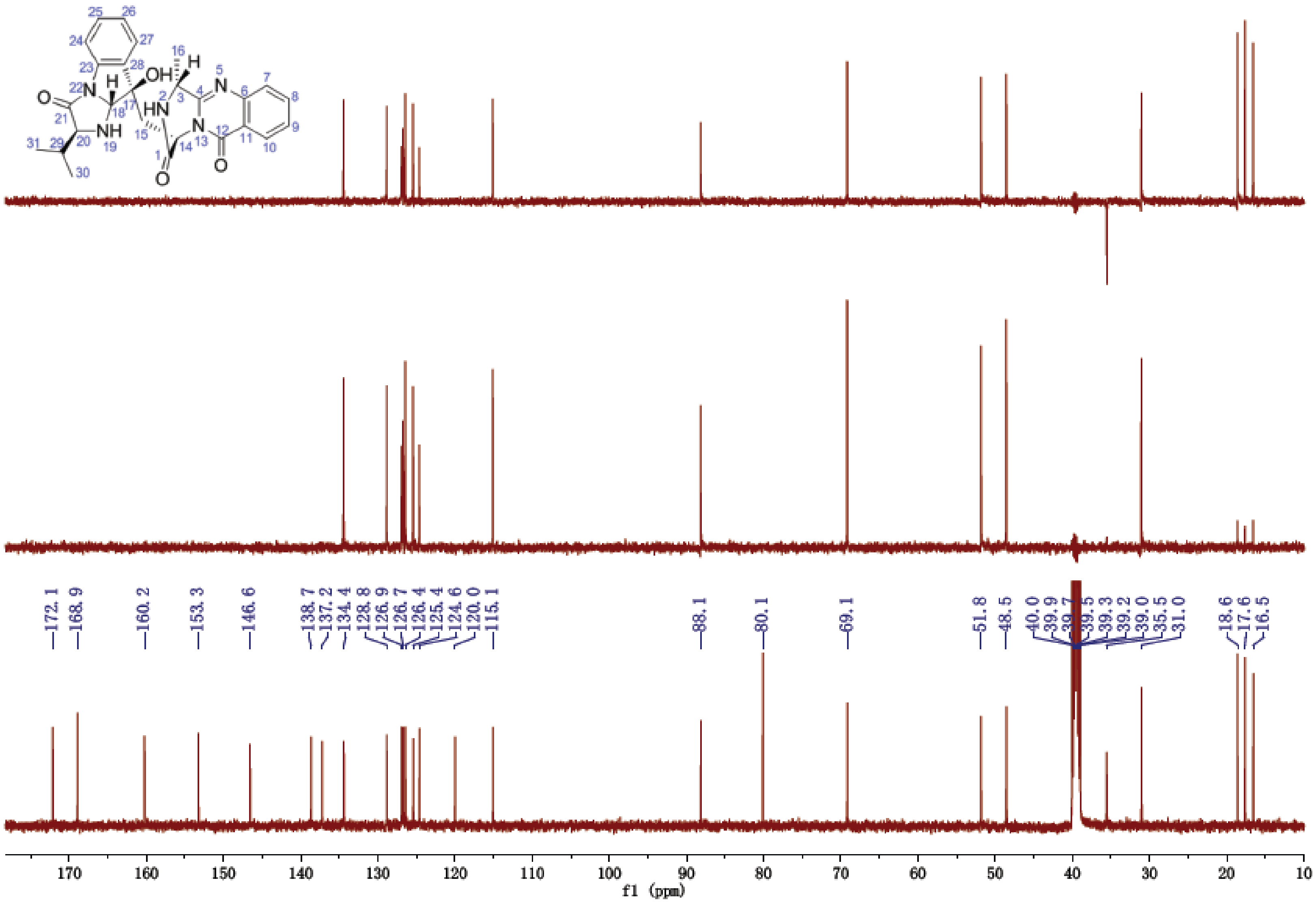

Figure S9. ^{1}H–^{1}H COSY spectrum of compound **2**.

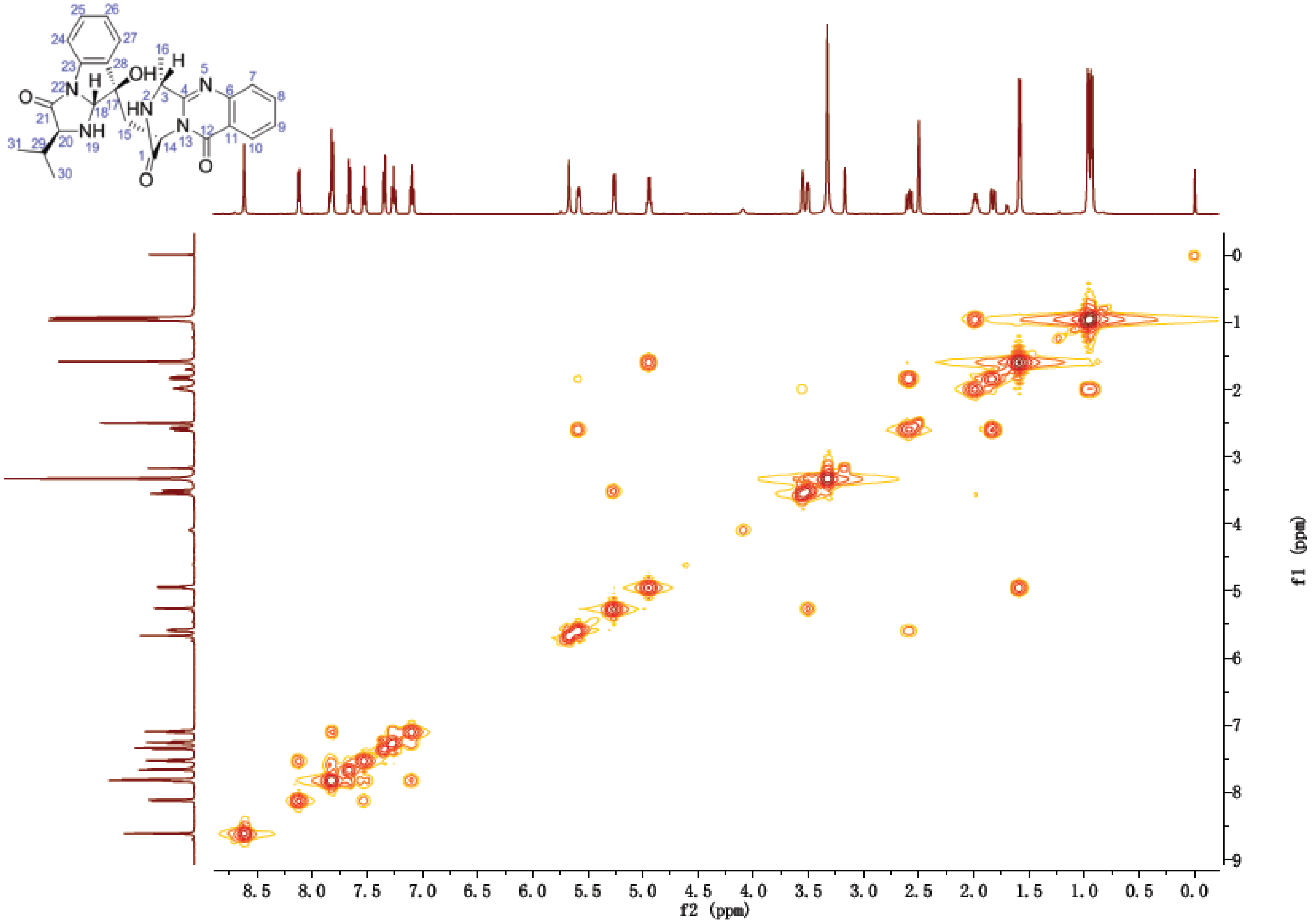

Figure S10. HSQC spectrum of compound **2**.

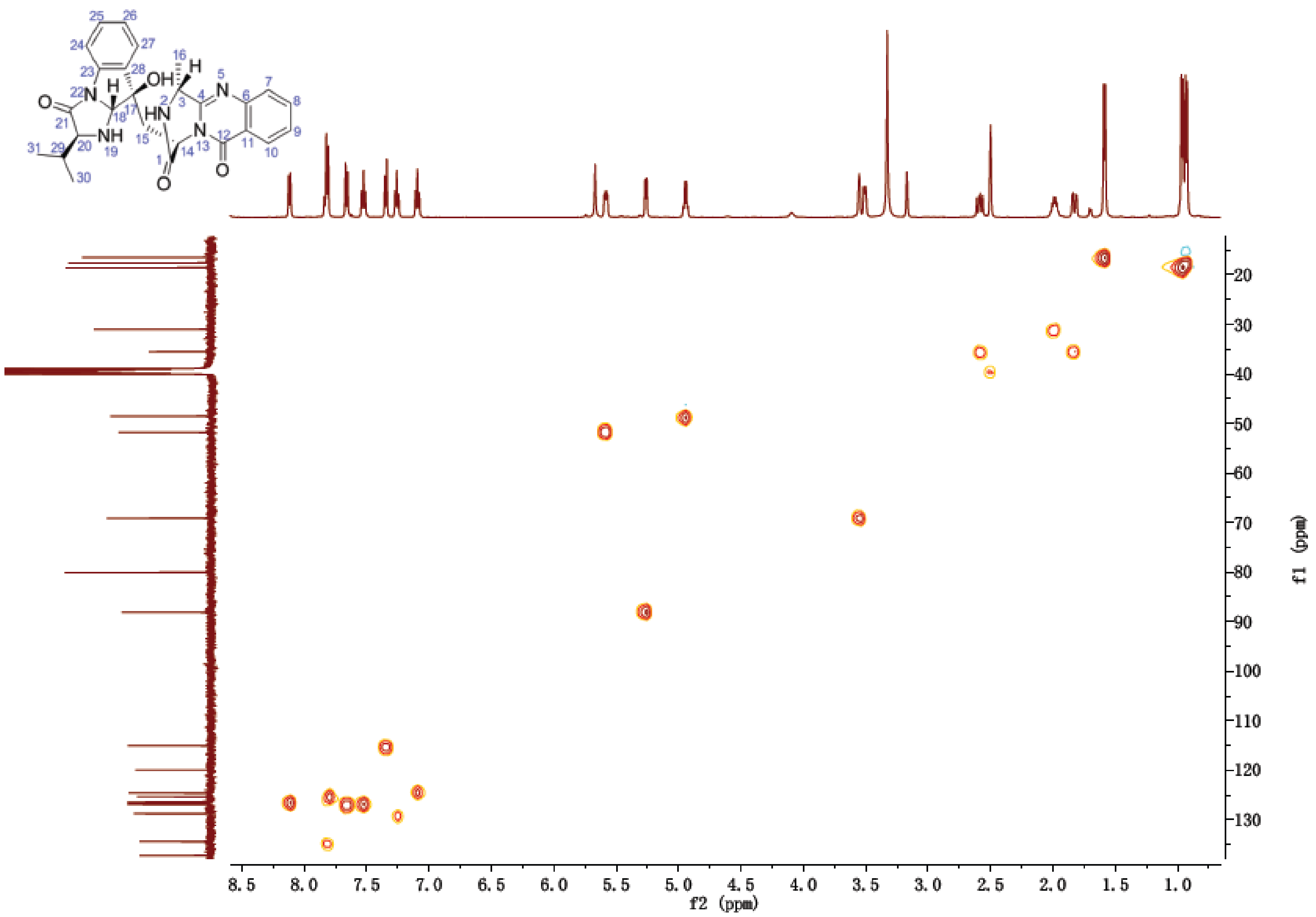

Figure S11. HMBC spectrum of compound **2**.

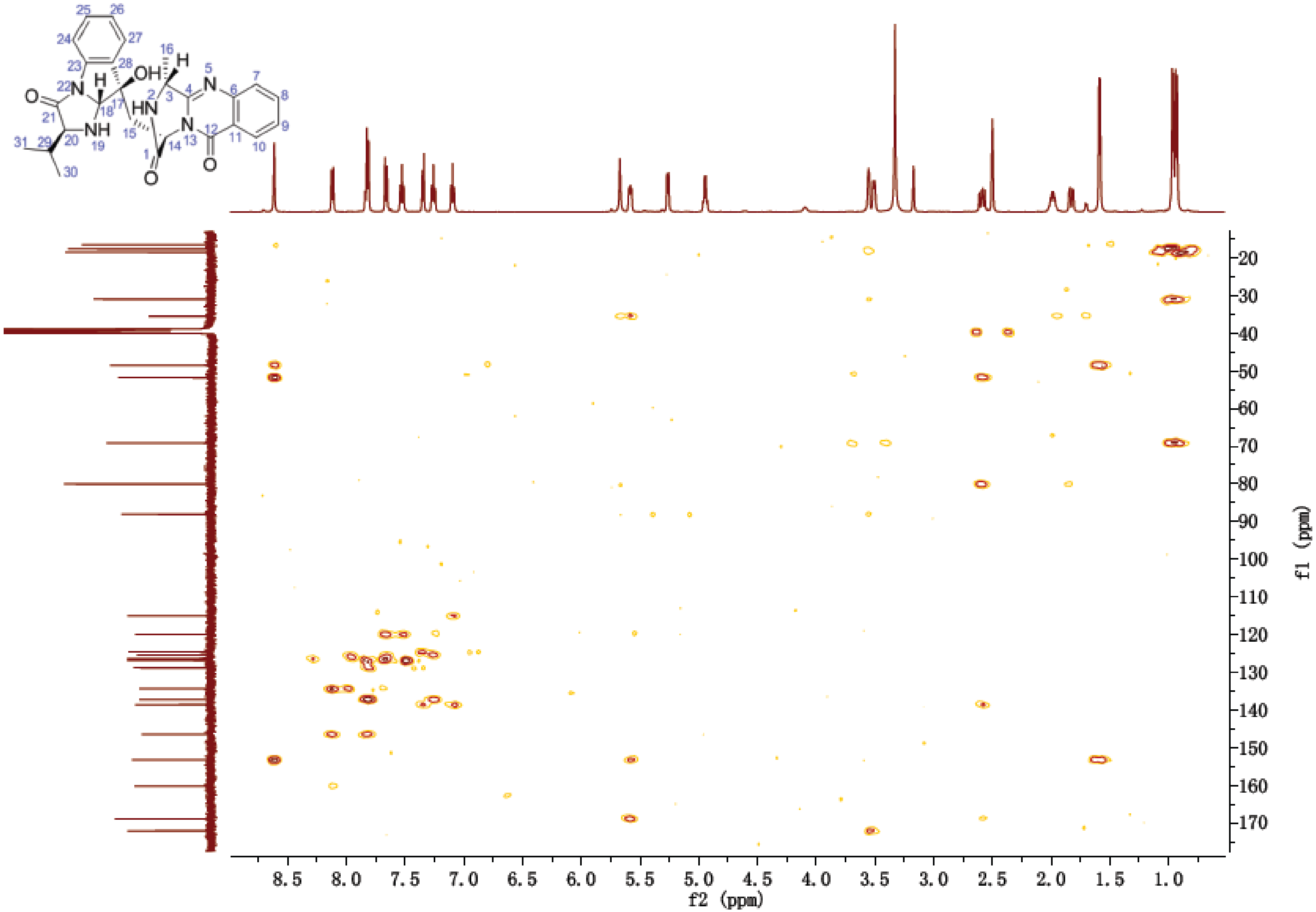

Figure S12a. NOESY spectrum of compound **2**.

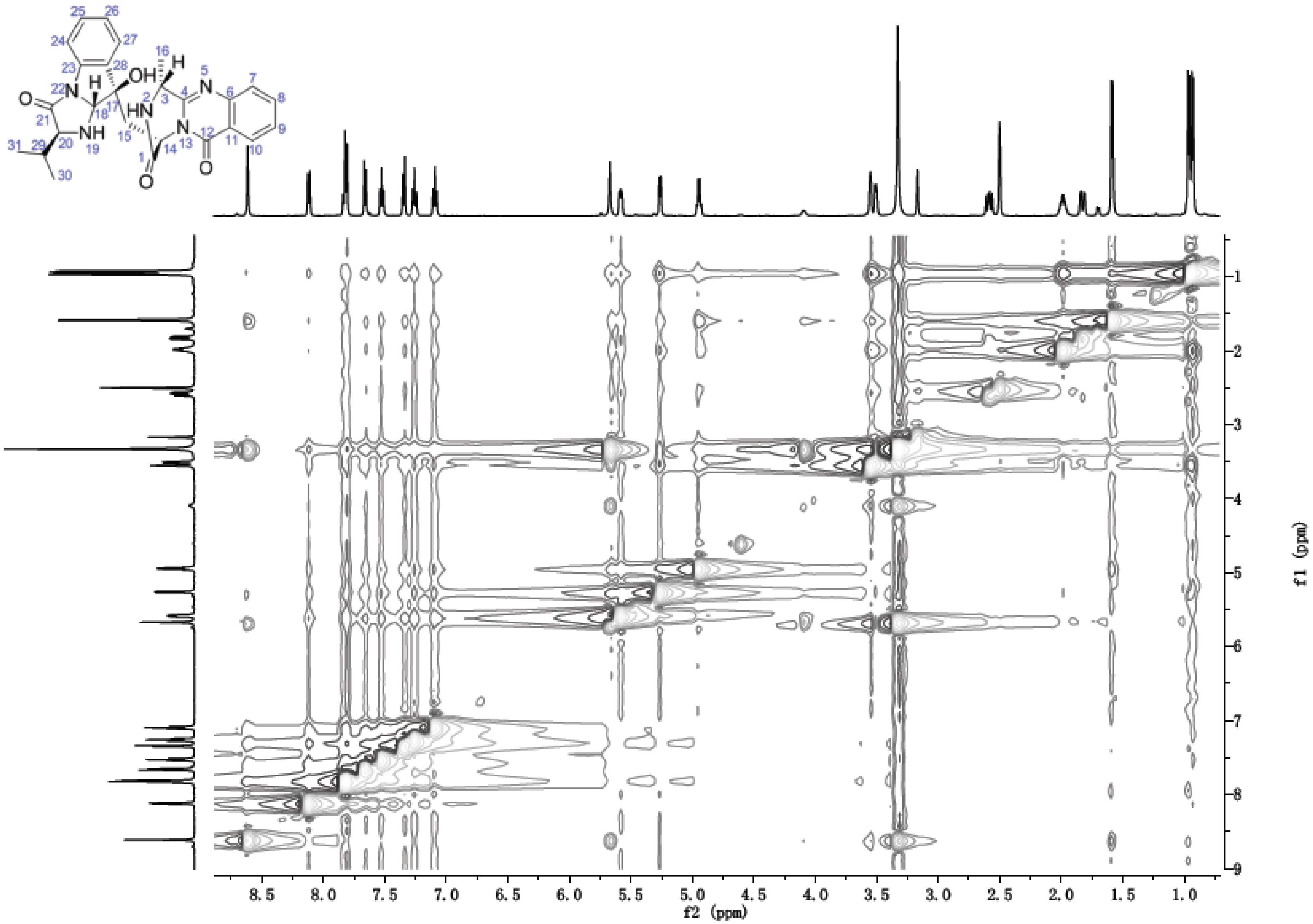

Figure S12b. an enlarged area of the NOESY spectrum of compound **2**.

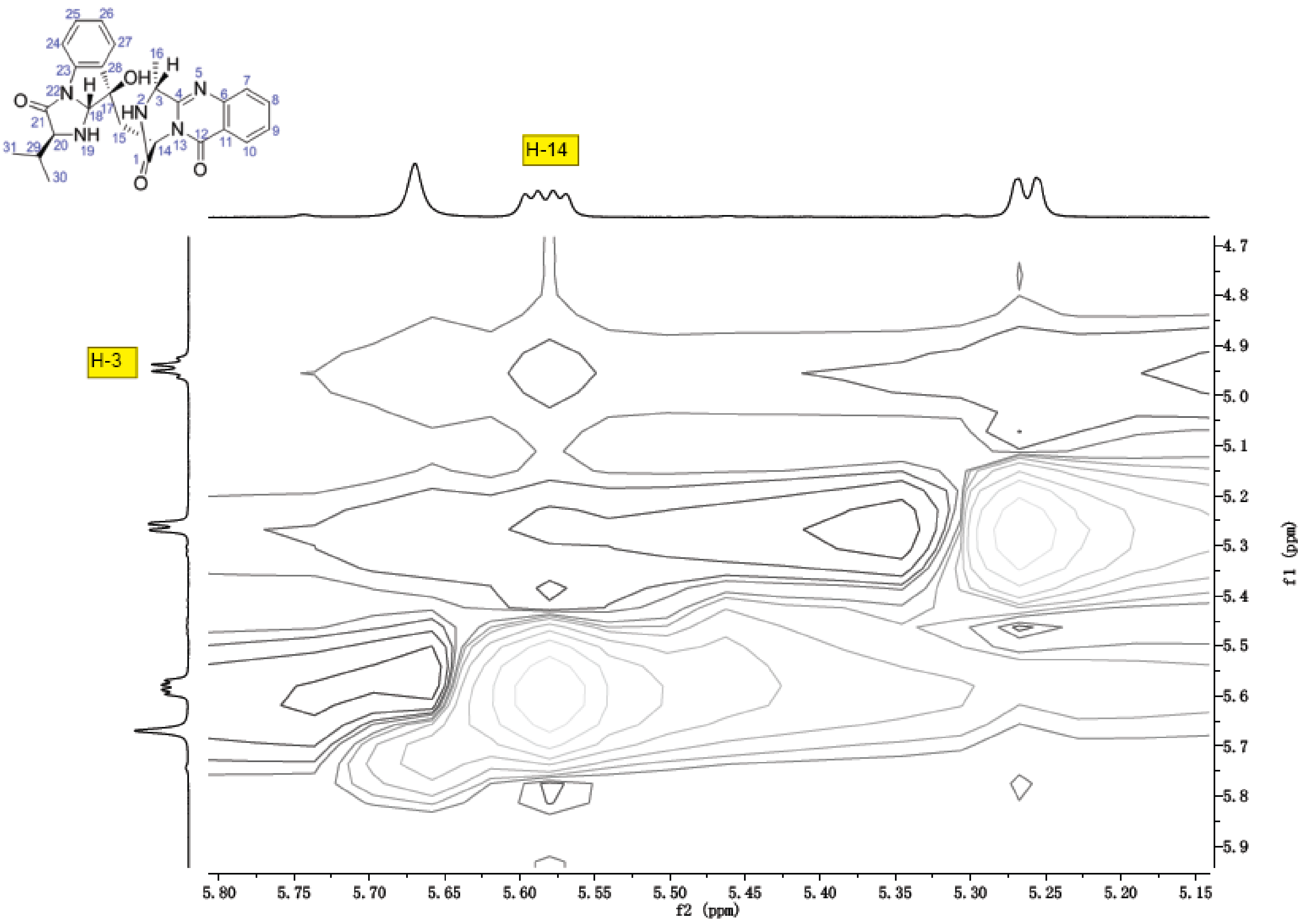

Figure S13. NOESY spectrum of compound **2** (recorded with different parameters).

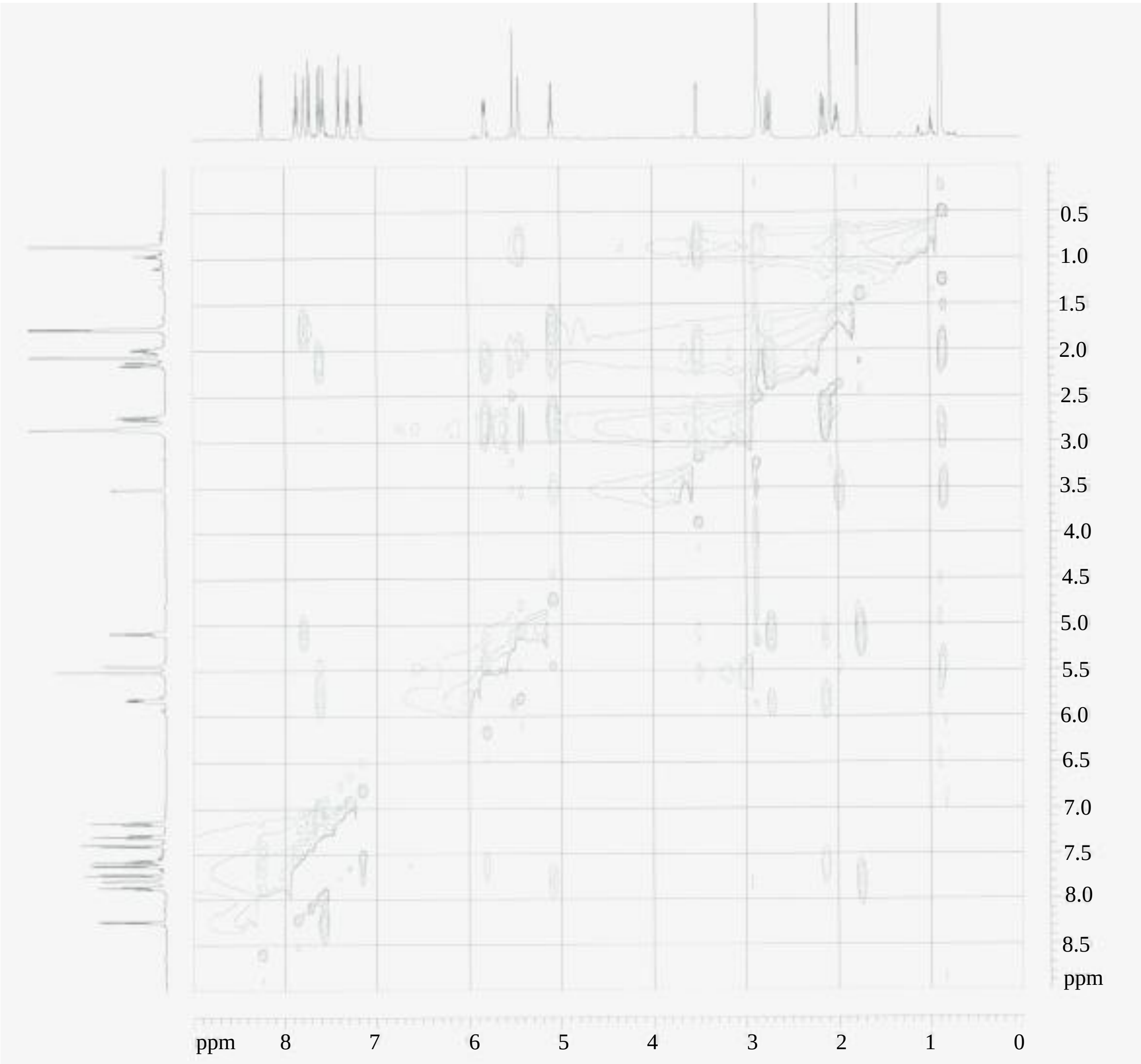

Figure S14. ^{1}H spectrum of compound **3**.

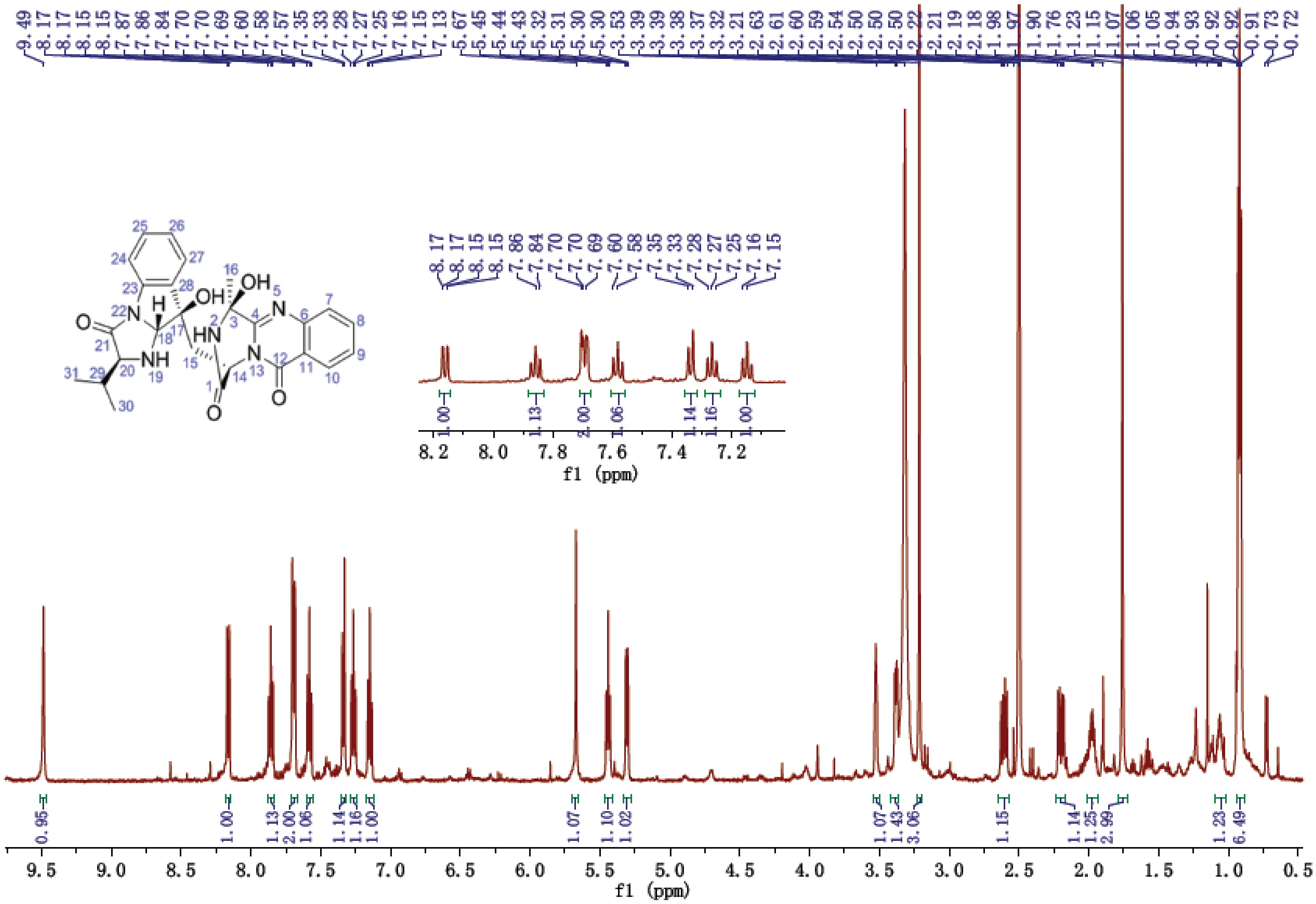

Figure S15. ^{13}C and DEPT spectra of compound **3**.

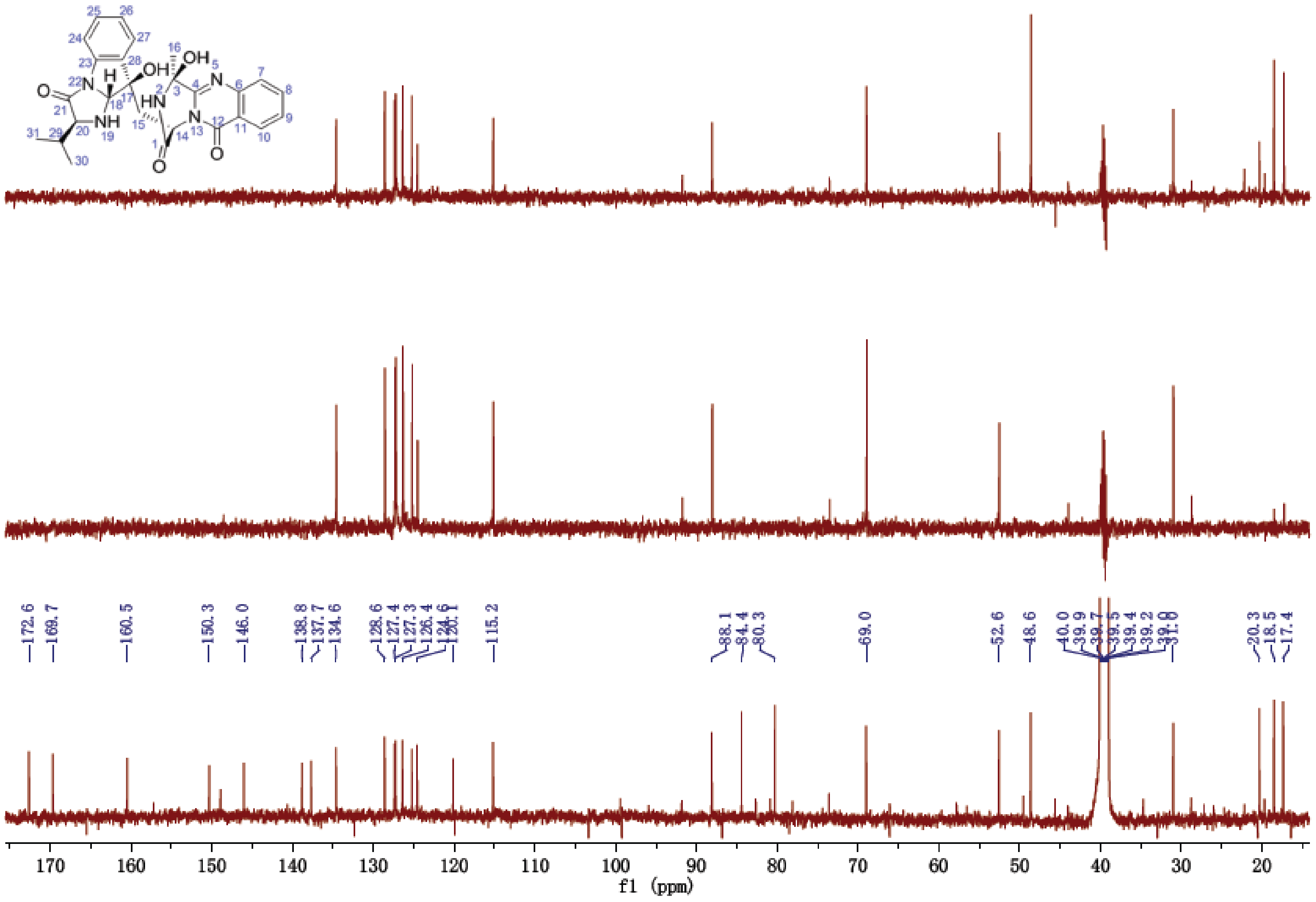

Figure S16. ^{1}H–^{1}H COSY spectrum of compound **3**.

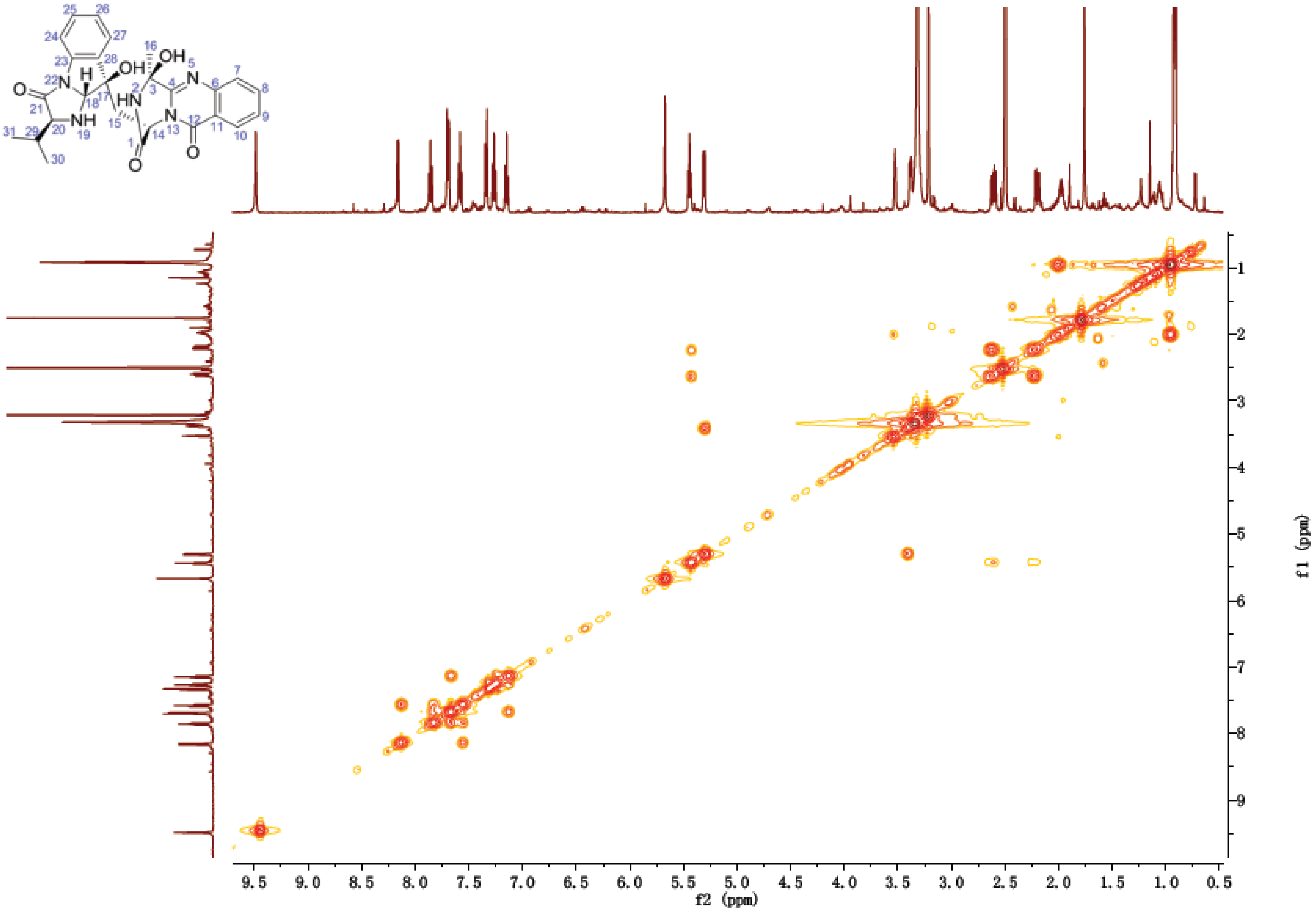

Figure S17. HSQC spectrum of compound **3**.

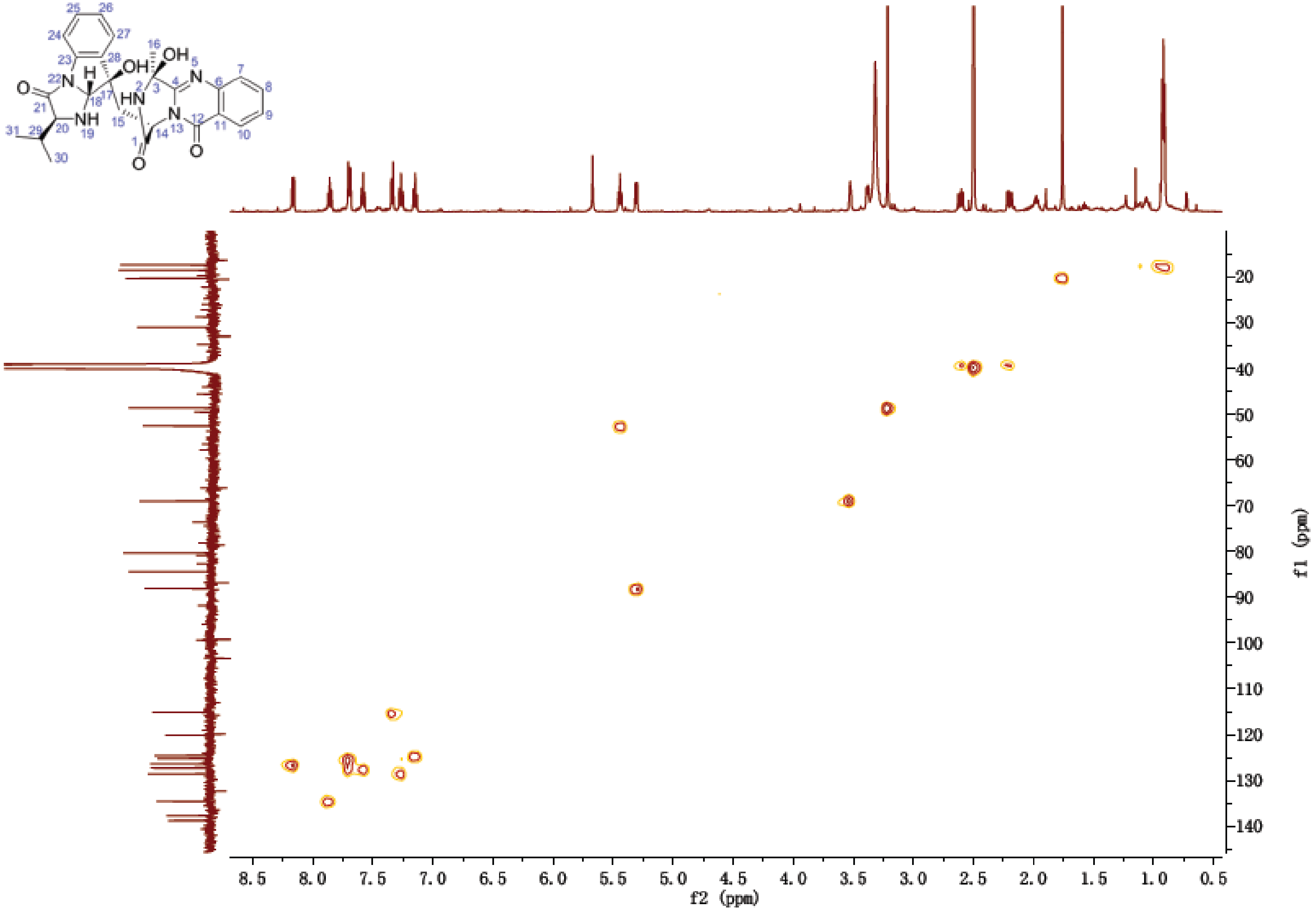

Figure S18. HMBC spectrum of compound **3**.

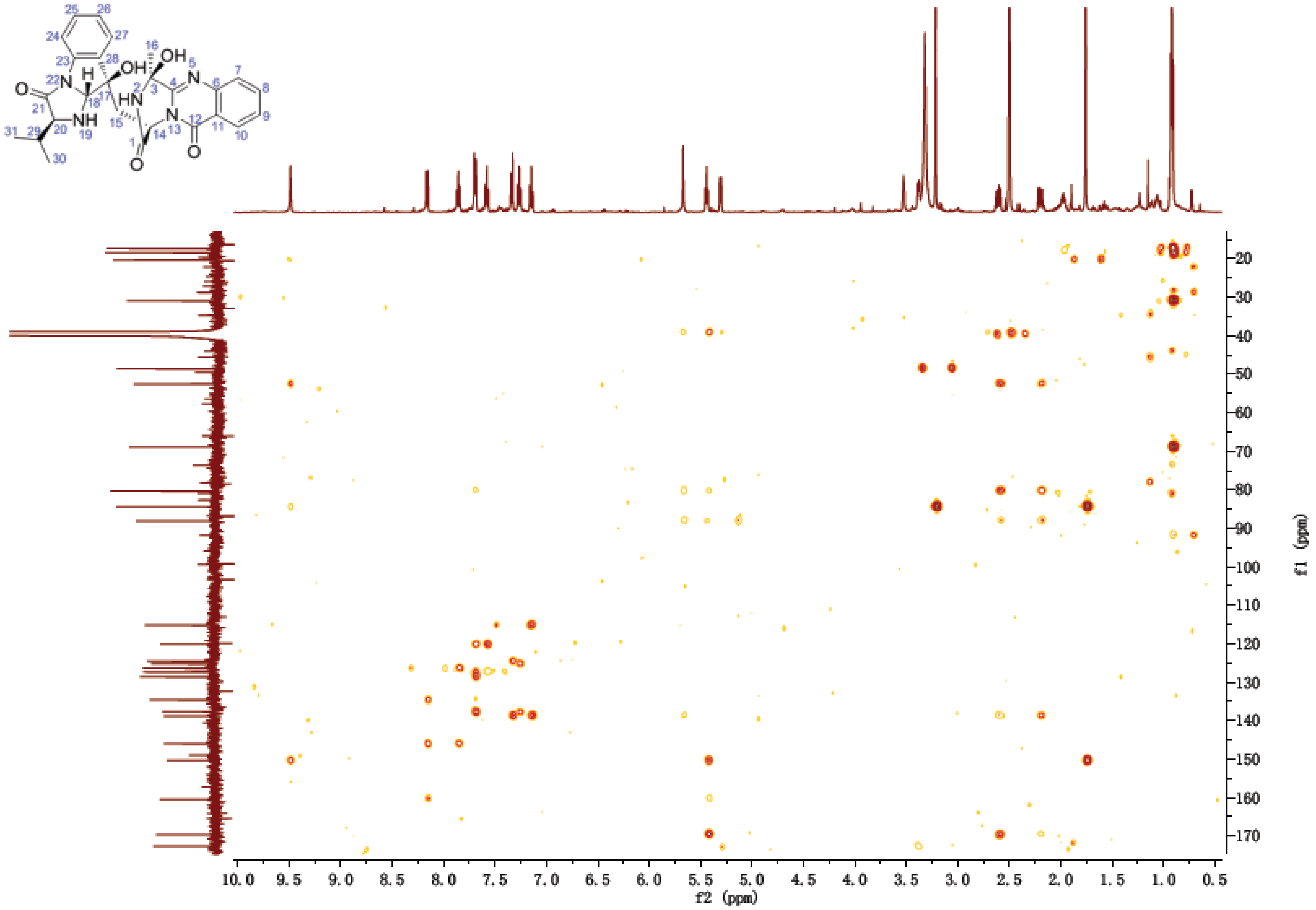

Figure S19. NOESY spectrum of compound **3**.

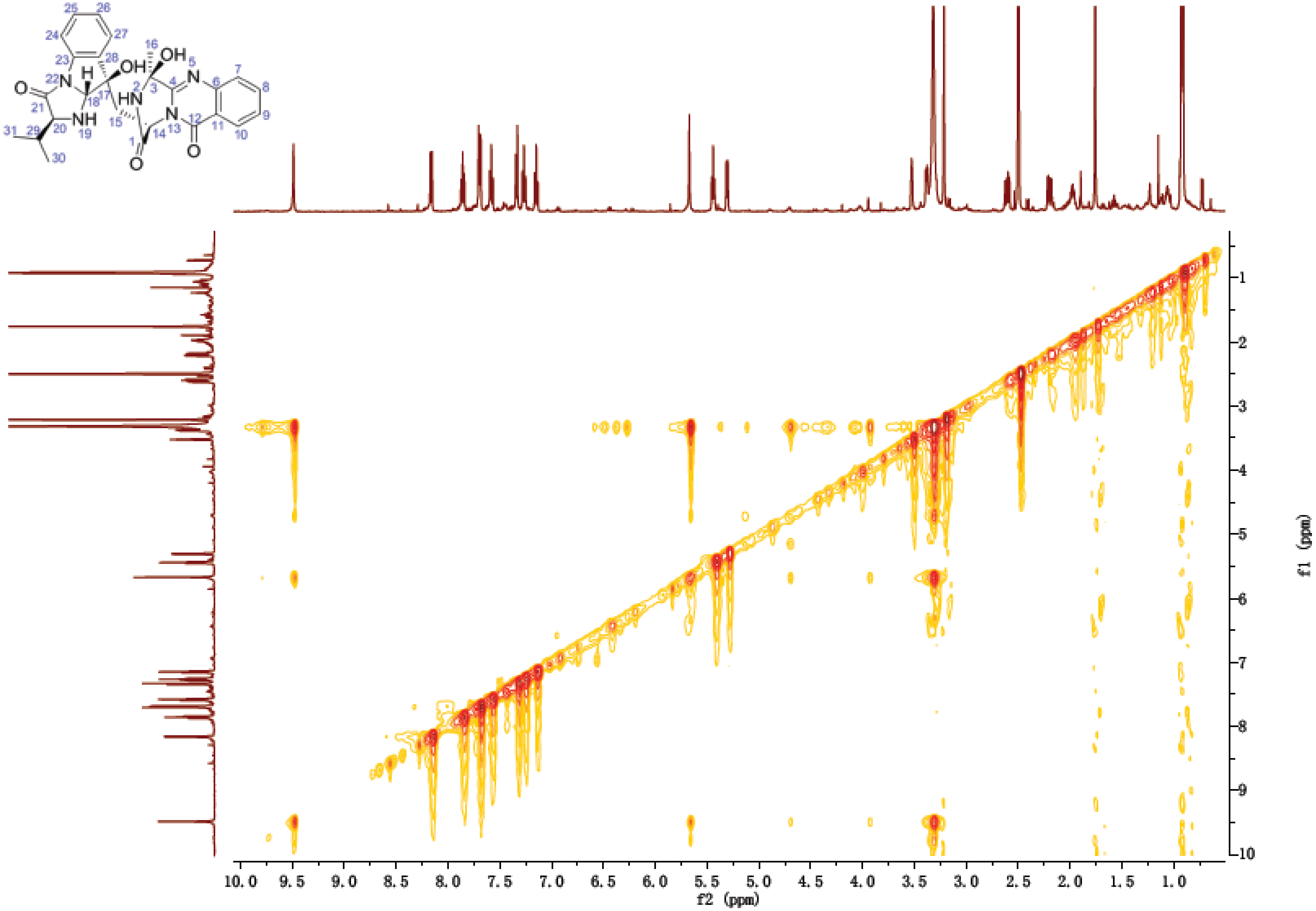

Figure S20. NOESY spectrum of compound **3** (recorded with different parameters).

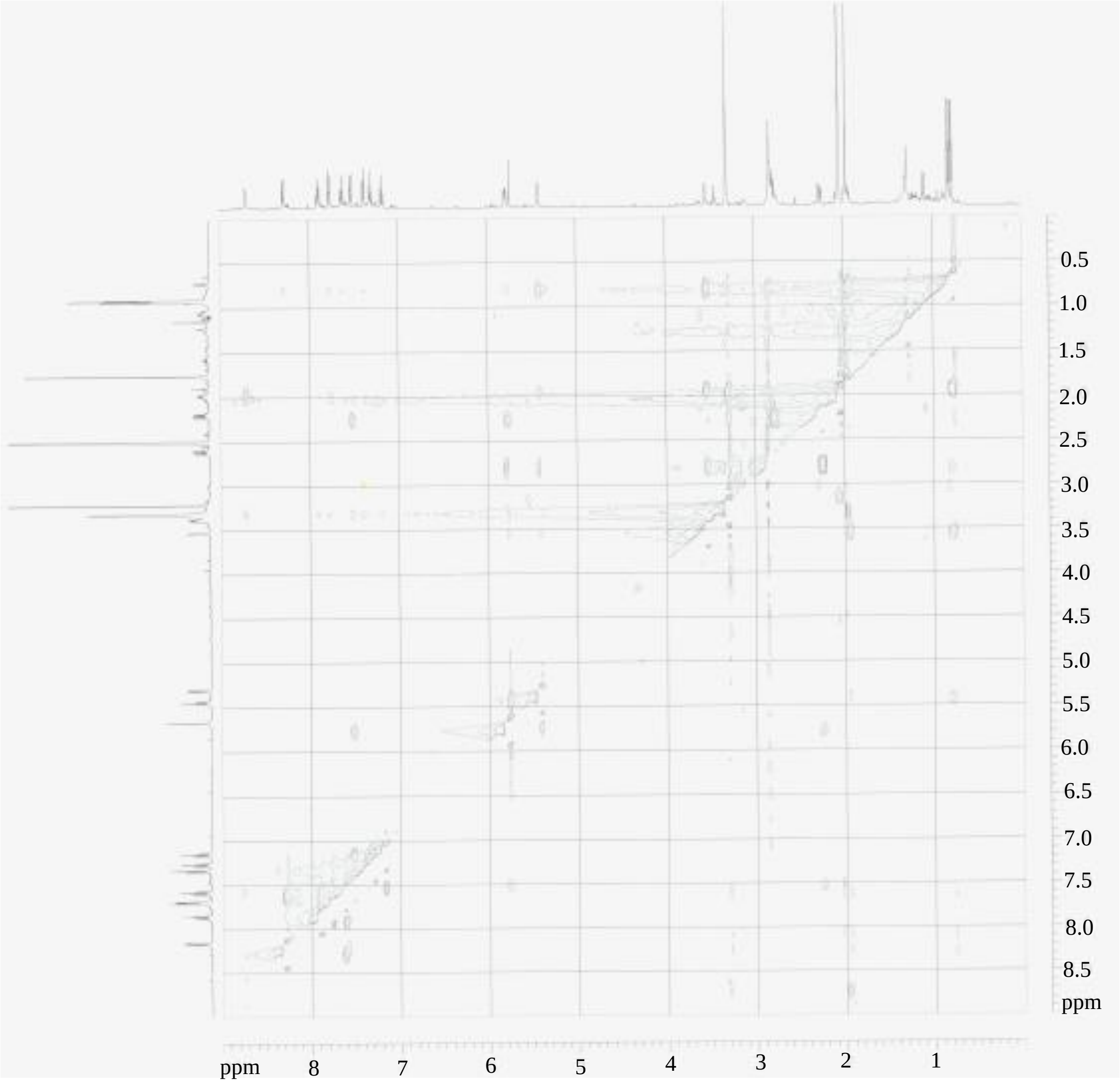

Figure S21. NOESY spectrum of compound **3** (recorded with different parameters).

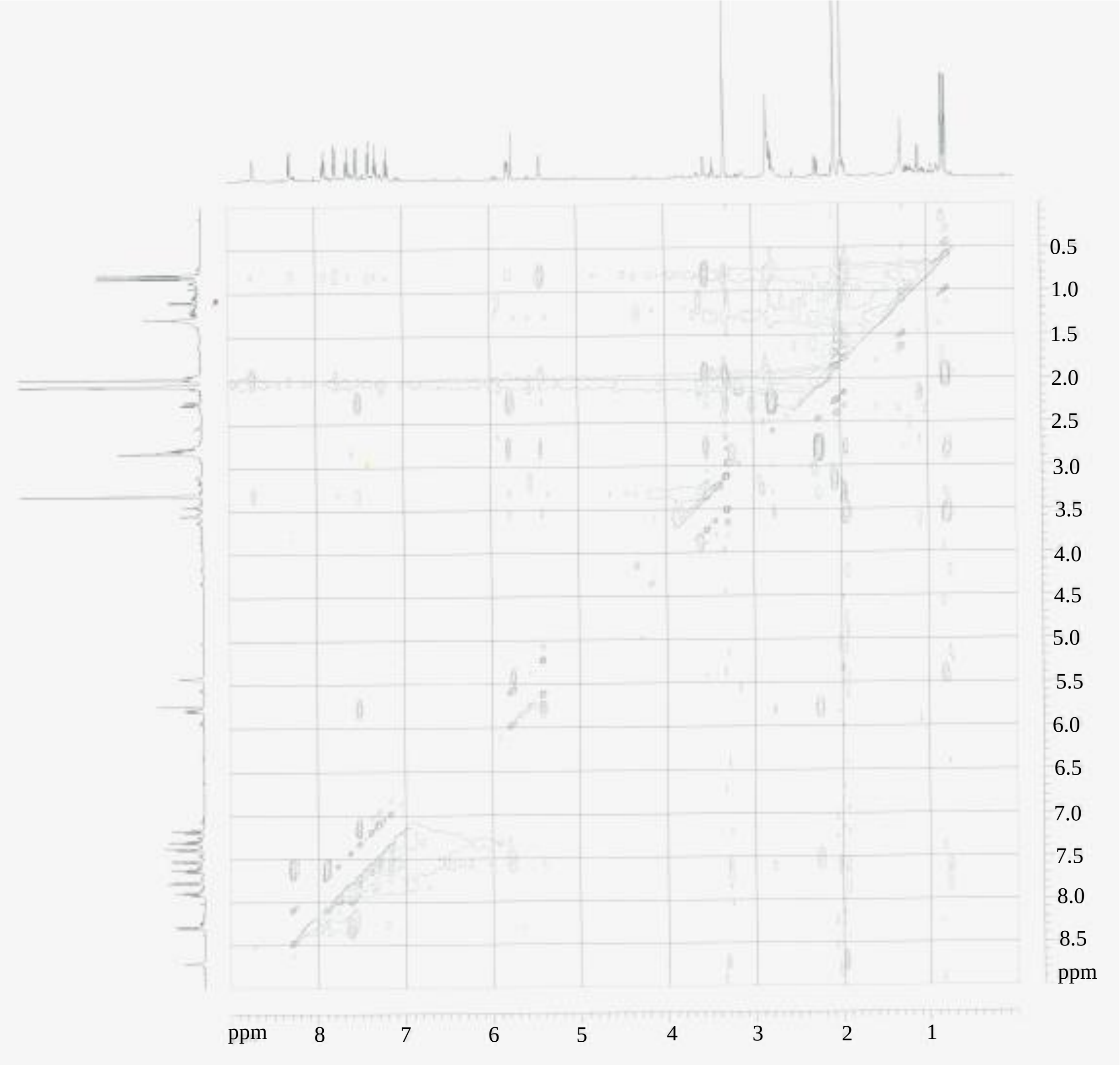

Figure S22. ^{1}H spectrum of compound **4**.

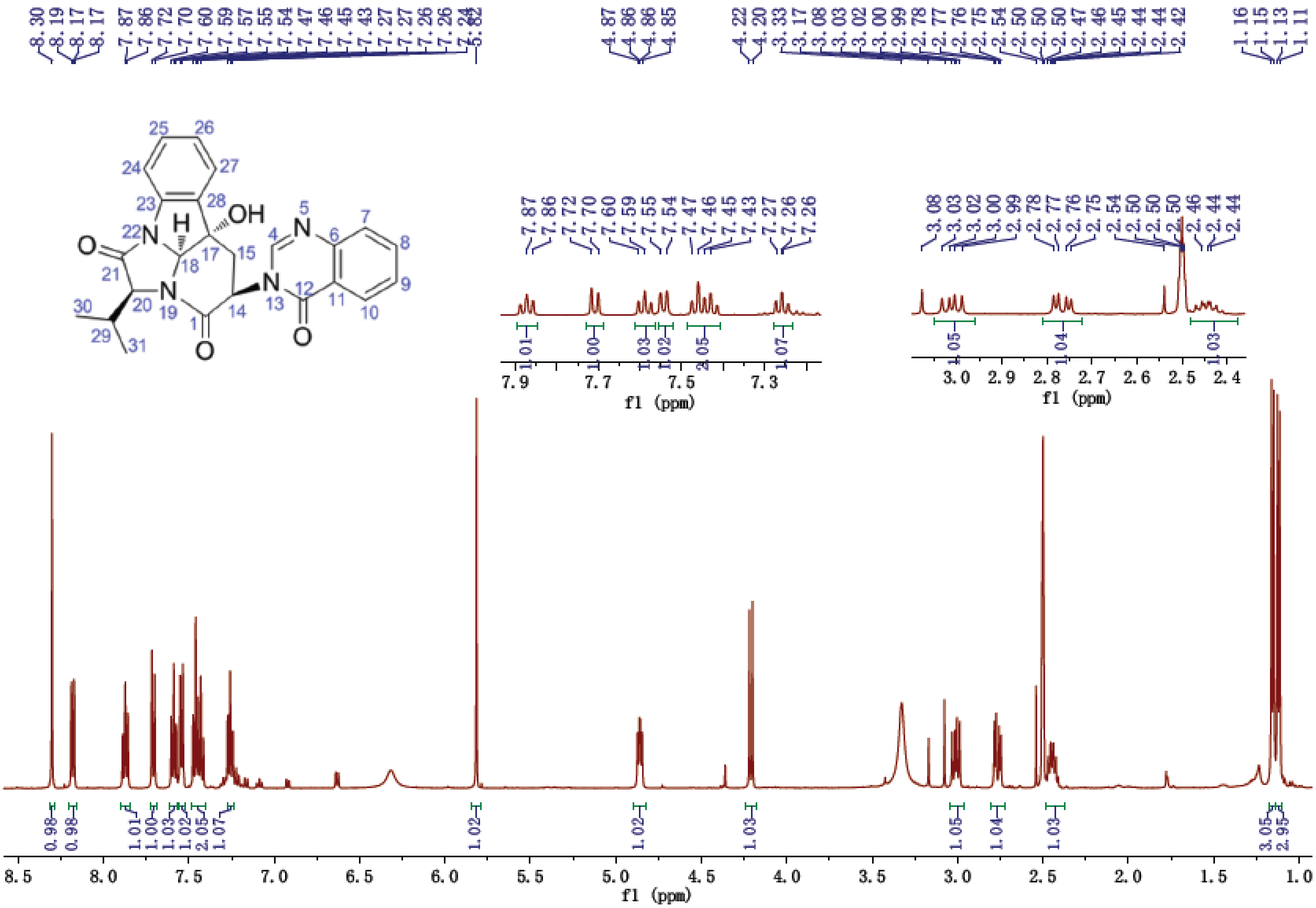

Figure S23. ^{13}C and DEPT spectra of compound **4**.

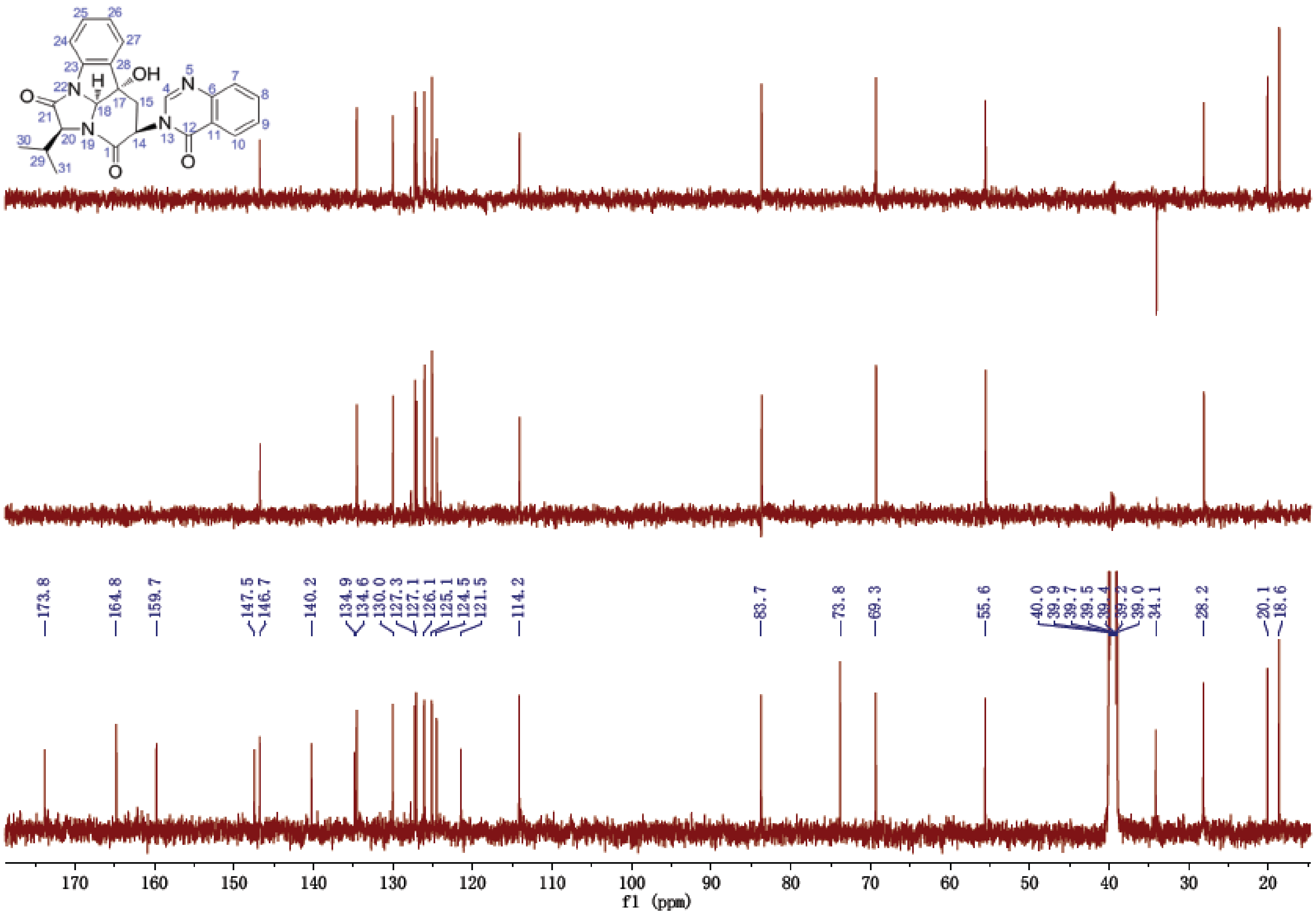

Figure S24. ^{1}H–^{1}H COSY spectrum of compound **4**.

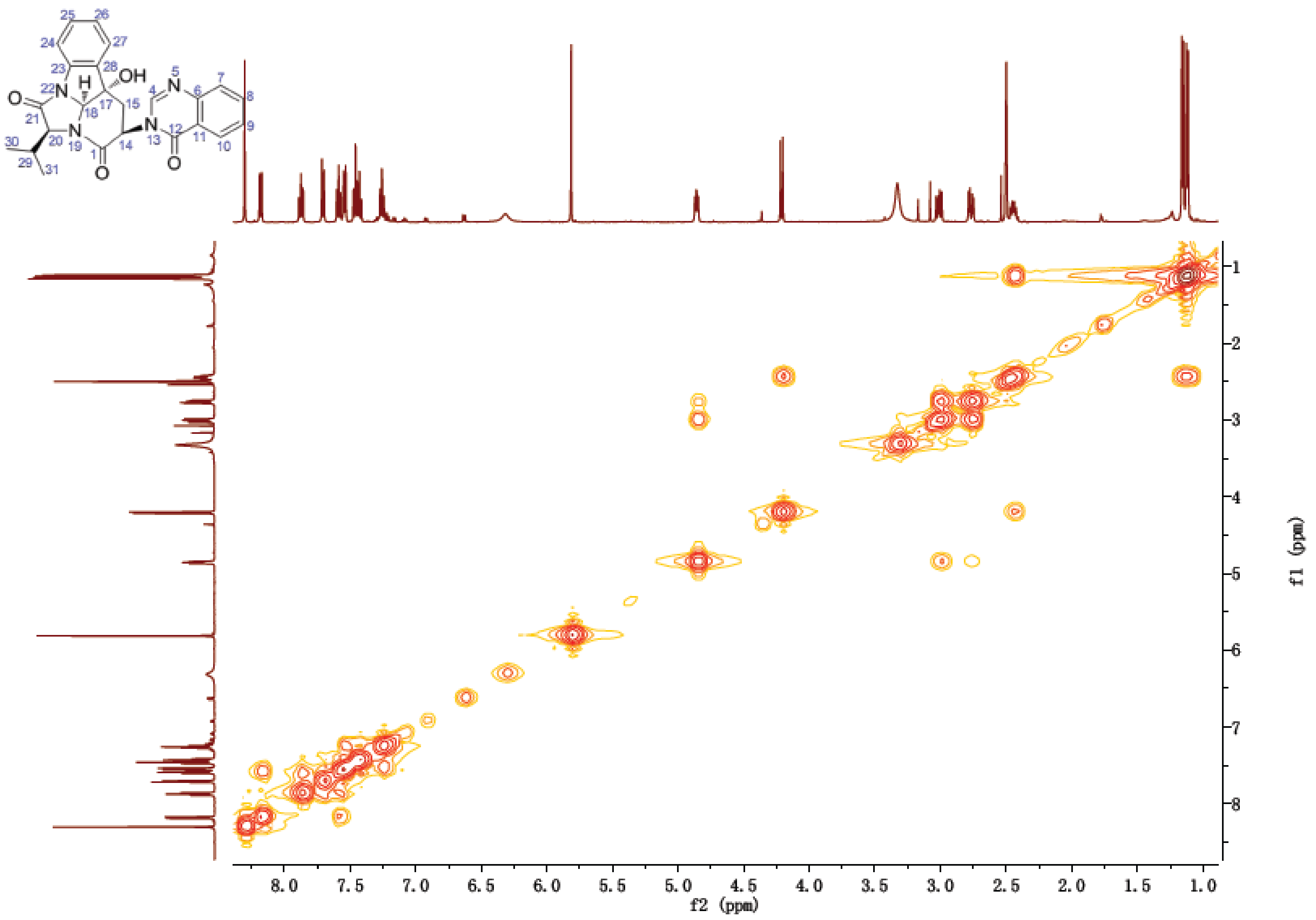

Figure S25. HSQC spectrum of compound **4**.

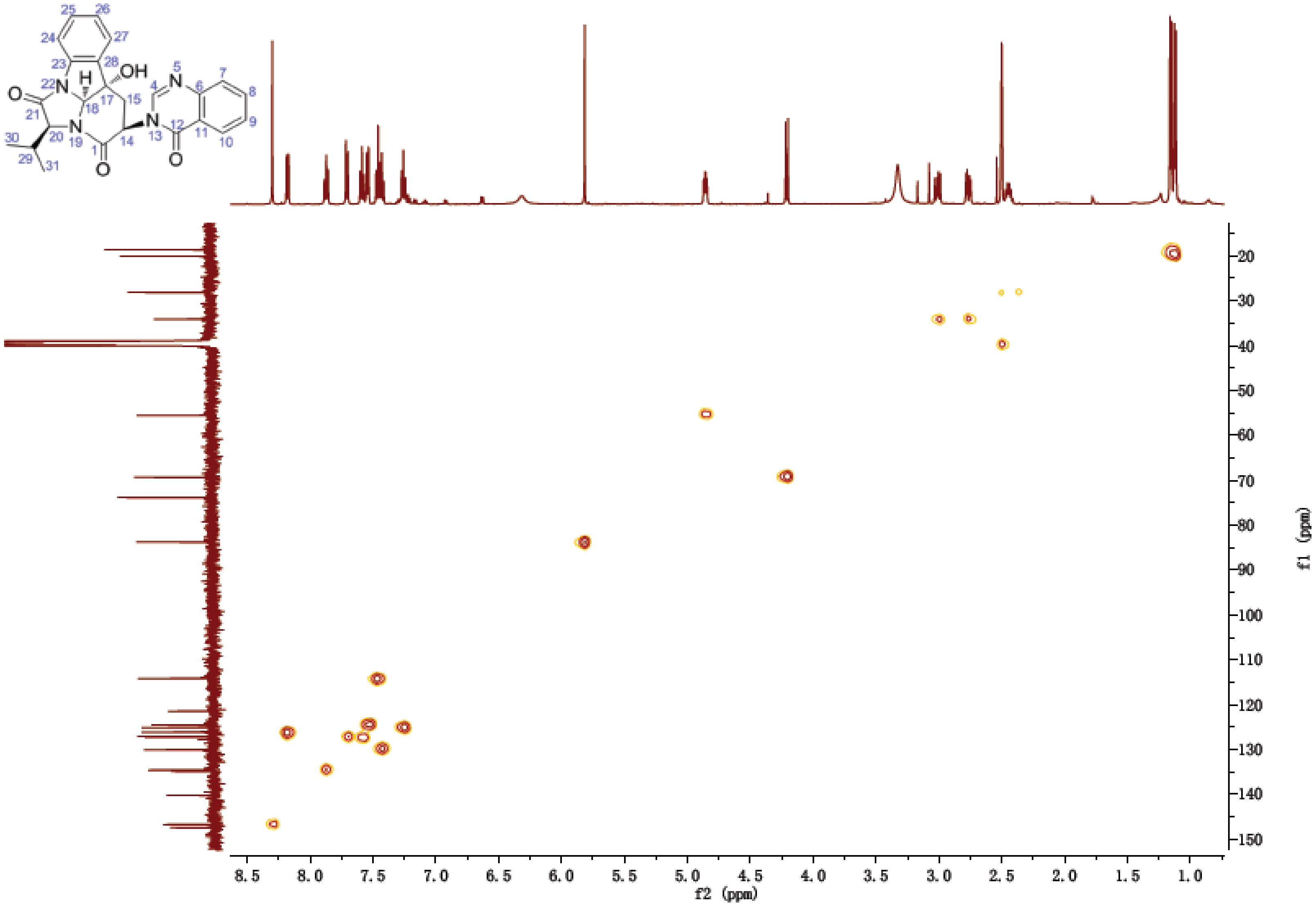

Figure S26. HMBC spectrum of compound **4**.

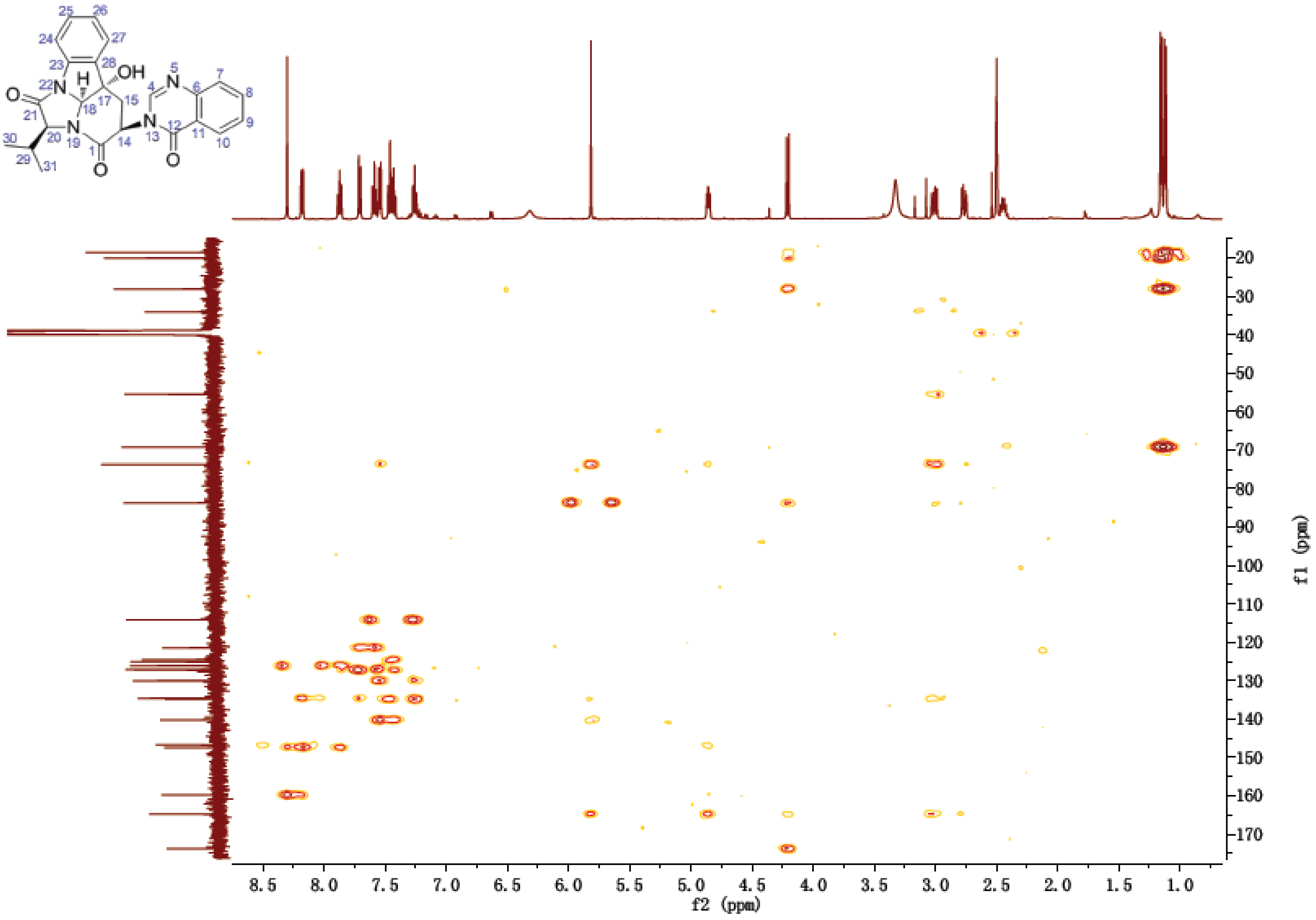

Figure S27. NOESY spectrum of compound **4**.

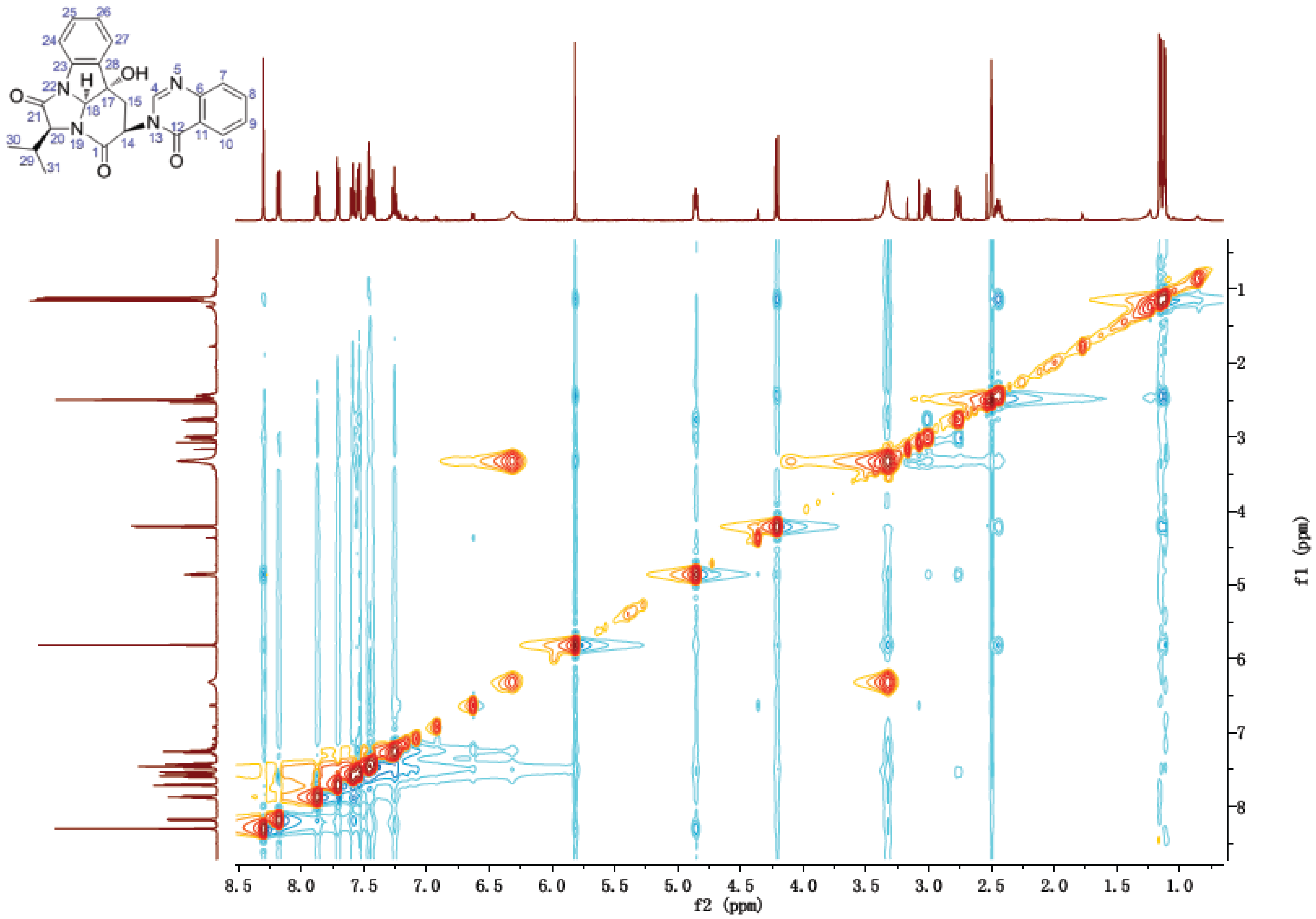

Figure S28. ^{1}H spectrum of the reducing product of compound **1** by $NaBH_4$ (measured in acetone-d_6).

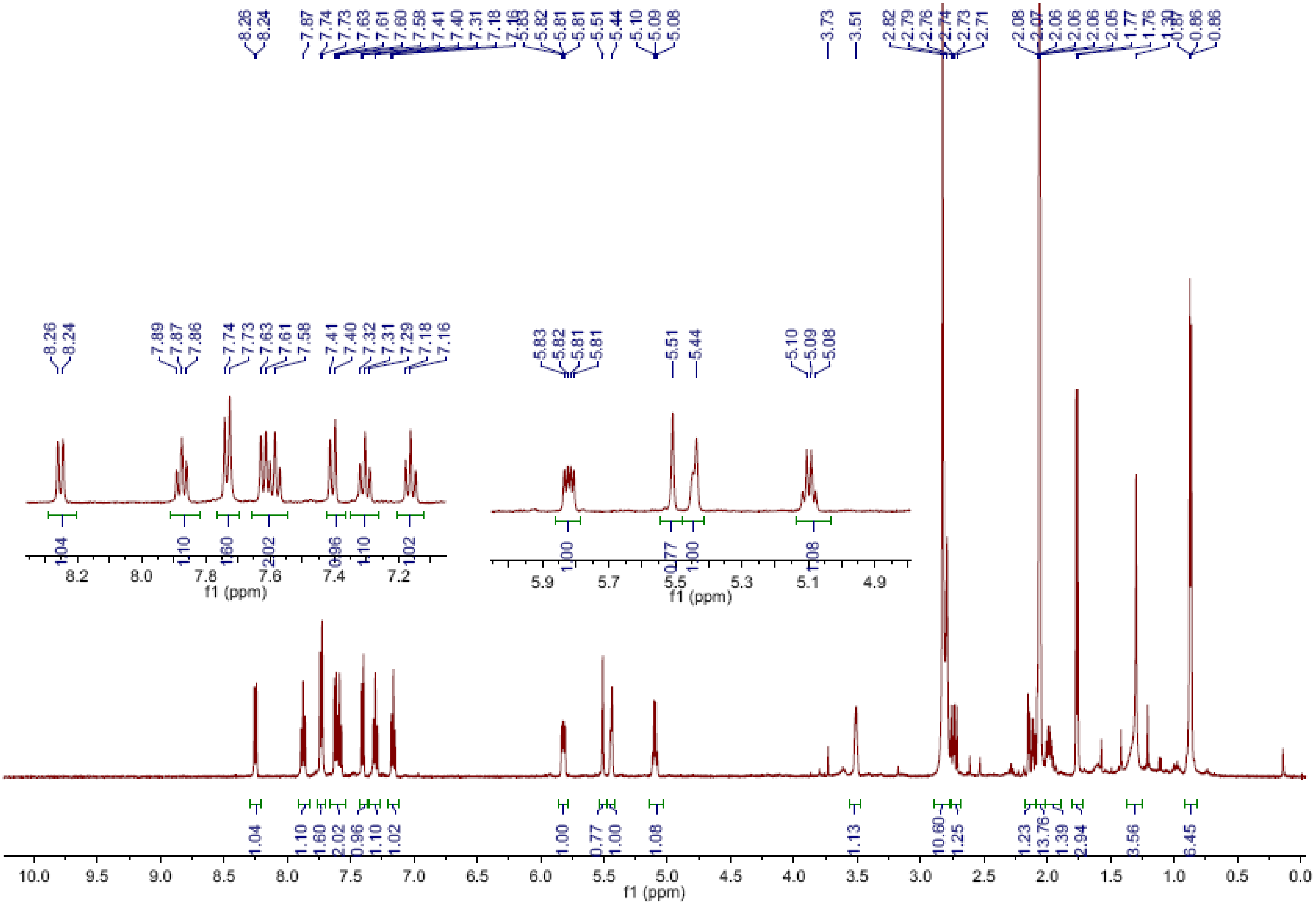

Figure S29. ^1H–^1H COSY spectrum of the reducing product of compound **1** by $NaBH_4$ (measured in acetone-d_6).

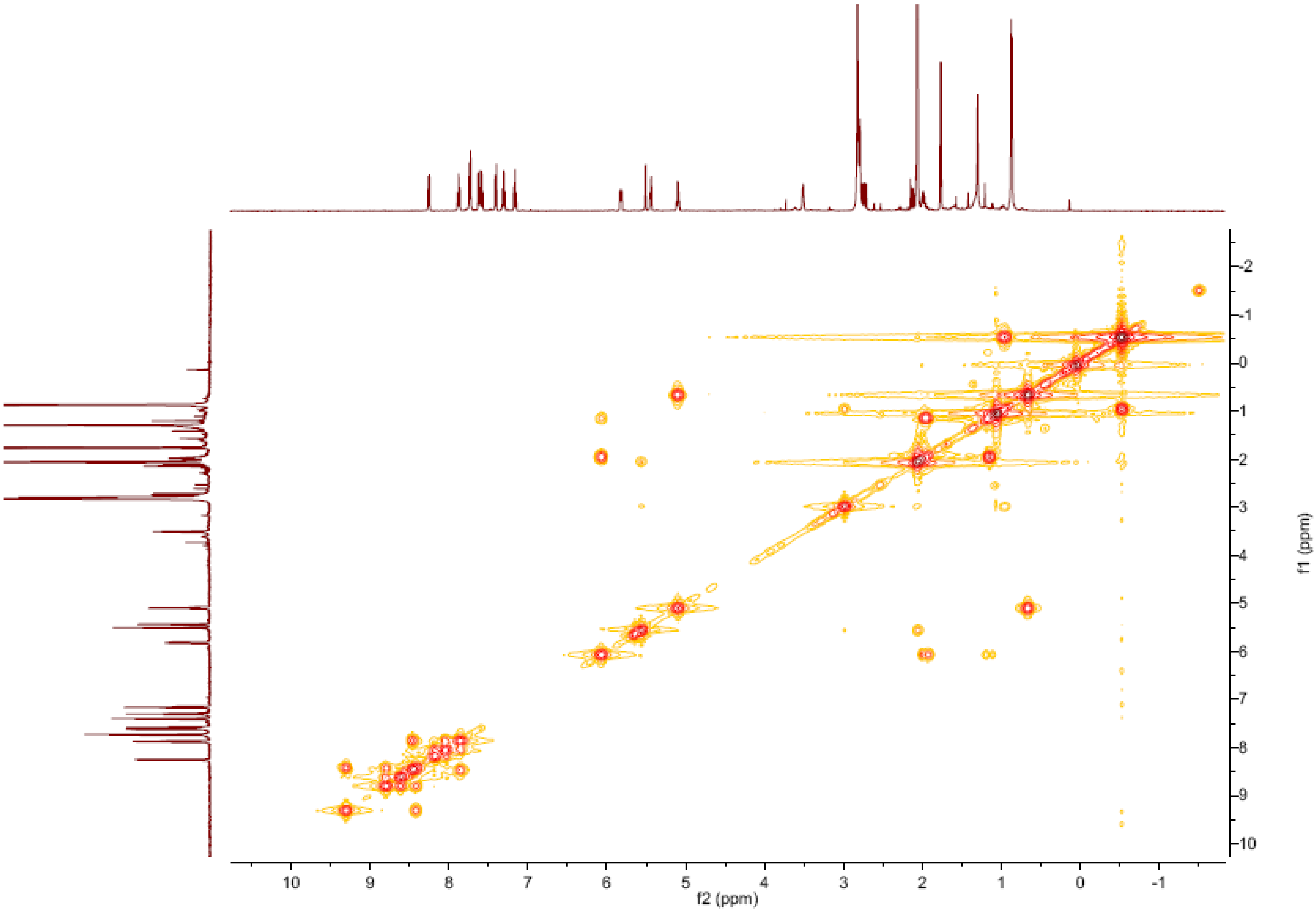

Figure S30. NOESY spectrum of the reducing product of compound **1** by $NaBH_4$ (measured in acetone-d_6).

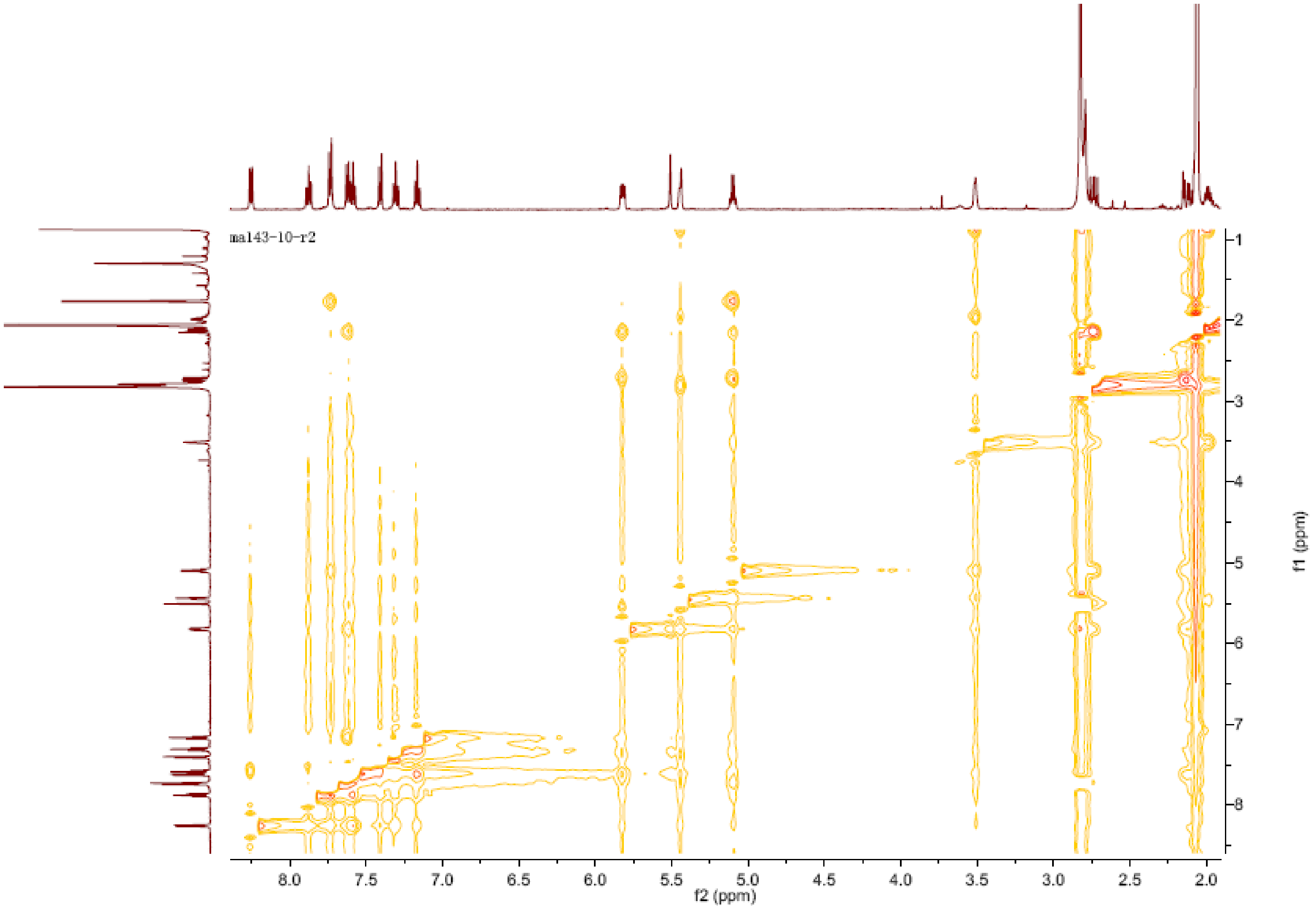

Supplement: Supplementary File 1 — Supplementary Information (PDF, 1503 KB) [file marinedrugs-11-02682-s001.pdf]
